# Red-Fleshed Apples Rich in Anthocyanins and White-Fleshed Apples Modulate the Aorta and Heart Proteome in Hypercholesterolaemic Rats: The AppleCOR Study

**DOI:** 10.3390/nu14051047

**Published:** 2022-02-28

**Authors:** Úrsula Catalán, Anna Pedret, Silvia Yuste, Laura Rubió, Carme Piñol, Berner Andrée Sandoval-Ramírez, Judit Companys, Elisabet Foguet, Pol Herrero, Núria Canela, Maria-Jose Motilva, Rosa Solà

**Affiliations:** 1 Functional Nutrition, Oxidation, and CVD Research Group (NFOC-Salut), Medicine and Surgery Department, Faculty of Medicine and Health Sciences, Universitat Rovira i Virgili, 43201 Reus, Spain; ursula.catalan@urv.cat (Ú.C.); andreesandoval1@gmail.com (B.A.S.-R.); rosa.sola@urv.cat (R.S.); 2 Institut d’Investigació Sanitària Pere Virgili (IISPV), 43204 Reus, Spain; 3 Unitat de Nutrició i Salut, Eurecat, Centre Tecnològic de Catalunya, 43204 Reus, Spain; judit.companys@eurecat.org; 4 Food Technology Department, Universitat de Lleida-AGROTECNIO Center, 25198 Lleida, Spain; silvia.yuste@udl.cat (S.Y.); laura.rubio@udl.cat (L.R.); 5 Department of Medicine, Universitat de Lleida, 25008 Lleida, Spain; carme.pinyol@udl.cat; 6 Institut de Recerca Biomèdica de Lleida, Fundació Dr. Pifarré-IRBLleida, 25198 Lleida, Spain; 7 Eurecat, Centre Tecnològic de Catalunya, Centre for Omic Sciences (COS), Joint Unit Universitat Rovira i Virgili-EURECAT, 43204 Reus, Spain; elisabet.foguet@eurecat.org (E.F.); pol.herrero@eurecat.org (P.H.); nuria.canela@eurecat.org (N.C.); 8 Instituto de Ciencias de la Vid y del Vino (ICVV), Gobierno de La Rioja, CSIC, Universidad de La Rioja, 26007 Logroño, Spain; motilva@icvv.es; 9 Hospital Universitari Sant Joan de Reus (HUSJR), 43204 Reus, Spain

**Keywords:** anthocyanins, phenolic compounds, proteomics, aorta and heart rat tissues, apples

## Abstract

The impact of a red-fleshed apple (RFA) rich in anthocyanins (ACNs), a white-fleshed apple (WFA) without ACNs, and an extract infusion from *Aronia* fruit (AI) equivalent in dose of cyanidin-3-O-galactoside (main ACN) as RFA was determined by the proteome profile of aorta and heart as key cardiovascular tissues. Hypercholesterolaemic Wistar rats were separated into six groups (*n* = 6/group; three males and three females) and the proteomic profiles were analyzed using nanoliquid chromatography coupled to mass spectrometry. No adverse events were reported and all products were well tolerated. RFA downregulated C1QB and CFP in aorta and CRP in heart. WFA downregulated C1QB and CFP in aorta and C9 and C3 in aorta and heart, among other proteins. AI downregulated PRKACA, IQGAP1, and HSP90AB1 related to cellular signaling. Thus, both apples showed an anti-inflammatory effect through the complement system, while RFA reduced CRP. Regardless of the ACN content, an apple matrix effect was observed that involved different bioactive components, and inflammatory proteins were reduced.

## 1. Introduction

Cardiovascular disease (CVD) remains a major cause of health loss throughout all regions of the world [1]. A key component of preventing CVD is a healthy lifestyle that includes frequent physical activity [2,3] and a healthy diet with high amounts of vegetables and fruits [4], including apples [4,5]. In particular, apples, due to their geographical distribution, organoleptic properties, and seasonal availability, are some of the most popular fruits consumed around the world [6], and their associations and effects on CVD have been described [7]. The apple components that can influence CVD include phenolic compounds (PCs), polysaccharides or fibre (pectin), phytosterols, pentacyclic triterpenes, vitamins and trace elements [8]. The level of apple PCs can range between 5230 and 27,240 mg/kg of the apple dry weight, and the most common PC types are hydroxycinnamic acids (50–3000 mg/kg), flavanols (4622–25,480 mg/kg), anthocyanins (ACNs; 10–551 mg/kg), and dihydrochalcones (49–434 mg/kg) [9]. Notwithstanding, phenolic patterns change across the different apple varieties and also change by the weather, season, geographical distribution, and maturity of the fruit at the time of harvest [10,11]. However, the mechanisms of action of apple PCs on CVD were involved in the insulin sensitizing effects observed in obese Zucker rats during a meal tolerance test [12]. Moreover, apple PCs upregulated the expression of different antioxidant proteins, such as glutathione peroxidase, catalase dismutase and superoxide dismutase, in the liver and upregulated the hepatic genes associated with PPARα and therefore provided a healthier metabolic profile in obese Zucker rats [12].

Recently, ACN content in apples has attracted scientific interest because ACNs are PCs from the flavonol subclass with beneficial effects on CVD in humans, such as reducing hyperlipidemia [13].

In the last few years, there has been an increasing interest in red-fleshed apples with enhanced content of ACN. In this sense, it has been observed that red-fleshed apples supplementation in mice reduced inflammatory biomarkers and beneficially modulated the colonic micriobiota [14]. Moreover, red-fleshed apple supplementation in rats has ben related with protective effects against colon carcinogenesis [15]. Furthermore, in humans, ACN rich red-fleshed apples has been observed effects on immune function compared to poor ACN apples, and these changes were potentially associated with differences in the faecal microbiota [16]. 

The emerging potential effects of red-fleshed apples as a novel ACN-rich fruit along with the differences reported in the PC bioavailability and bioactivity depending on the food matrix, substantiates the present research focused on the possible health benefits of red-fleshed apple.

However, despite the growing efforts to study the emerging health benefits of red-fleshed apples, the mechanisms by which their consumption can improve CVD are still unknown. Therefore, it is challenging to determine the effects and possible mechanisms of action induced by apple consumption, in particular, how the effects differ in apples rich in ACNs and apples without ACNs.

Thus, to explore the underlying mechanisms of apple consumption, tissue proteomic analysis offers new perspectives to investigate how apple intake could regulate protein expression to potentially protect against CVD. Based on the literature, we hypothesize that a red-fleshed apple rich in ACNs produces protective changes in proteins of the heart or aorta as key CVD tissues.

The present study is framed in the AppleCOR project aimed to advance in the knowledge of the potential health effects and mechanisms of action of ACN, particularly a red-fleshed apple rich in ACN, in the improvement of the cardiometabolic risk factors through clinical studies in hypercholesterolemic subjects and mechanistic studies in diet induced hypercholesterolemic rats. To elucidate the pathobiology of hypercholesterolaemia, animal models can mimic the pathology of human, and Wistar rats models are extensively used models and suitable for tissue collection to their larger average body mass [17].

Specifically, the present study aims to determine new mechanisms of action of a red-fleshed apple variety rich in ACNs, a white-fleshed apple variety without ACNs, and an extract infusion from *Aronia melanocarpa* fruit, that has the equivalent dose of cyanidin-3-O-galactoside (the main ACN) as the red-fleshed apple variety, on the proteome profile of the aorta and heart, which are key CVD tissues, in hypercholesterolaemic rats.

## 2. Materials and Methods

### 2.1. Preparation of the Supplemented Diets in Wistar Rats

To compare the ACN effects of apples, two different apple varieties were selected: (i) the red-fleshed “Redlove” apple variety, a new genotype naturally biofortified with ACN, and (ii) the white-fleshed Granny Smith apple variety. Additionally, to study the effects of ACNs without the possible interactions of the apple matrix, an extract infusion from *Aronia melanocarpa* fruit with the equivalent dose of cyanidin-3-O-galactoside (the main ACN) as the red-fleshed apple variety was selected. 

Both apple varieties were provided by NUFRI SAT (Mollerussa, Lleida, Spain) and had different phytochemical profiles, particularly for ACNs, as described in Supplementary Table S1. To prepare the different diets, freeze-dried apple flesh was used as previously described [14] to preserve the ACNs and the rest of the phenolic compounds. Briefly, the apple cores were removed, and the whole apples (with peel) were cut into 1 cm-sized cubes. Then, the apple cubes were frozen in liquid nitrogen, lyophilized on a 15 TELSTAR Lyophilizer (Lyobeta, Terrassa, Spain), immediately transferred to airtight plastic containers and refrigerated (2 °C) until use in the preparation of the supplemented diets. Moreover, the apples were defrosted every 3 days to maintain the stability of the ACNs and the rest of the phenolic compounds. To obtain the extract infusion from *Aronia*, a cold water infusion of an *Aronia melanocarpa* fruit powder (Aronia Pulver, BIOJOY, Nuremberg, Germany) was prepared, which had the equivalent dose of cyanidin-3-O-galactoside as the red-fleshed apple variety. The *Aronia* powder was mixed with distilled water (1:1 proportion), and the mixture was homogenized (Kinematica Polytron, Polytron Corporation, Montreal, QC, Canada) for 60 s. The resulting infusion was centrifuged (5403× *g* for 5 min at room temperature), and the supernatant was analysed, filtered, stored in opaque containers protecting the phenolic compounds from light and frozen at −20 °C until use. Finally, this filtered *Aronia* extract infusion was added to the drinking water of the rats in opaque bottles. Every 3 days, drinking water was replaced and administered to the rats, adjusting the administered dose of cyanidin-3-O-galactoside according to the amount of water that the rats had drank. The phenol characterization of the freeze-dried apple flesh and the *Aronia* extract infusion is shown in Supplementary Table S1.

### 2.2. Animals and Experimental Procedure

As previously described [15], thirty-six Wistar rats weighing between 300 and 350 g were purchased from Charles River Laboratories (Barcelona, Spain). The rats were divided into six groups of six animals each (three males and three females) as follows: Group 1: standard chow diet (SCD) (2014, rodent maintenance diet, Envigo, Huntingdon, UK); Group 2: high-fat diet (HFD) (Atherogenic Rodent Diet TD. 02028, Envigo) to induce hypercholesterolemia; Group 3: HFD + red-fleshed apple (HFD + R); Group 4: HFD + white-fleshed apple (HFD + W), Group 5: HFD + *Aronia* extract infusion (HFD + A); and Group 6: HFD + atorvastatin (HFD + Atorv) as a hypolipidemic drug.

In this study, male and female rats were included to investigate the possibility of different biological effects because of sex. Group 1 was fed a chow diet for 9 weeks (Figure S1). The other groups were fed for 3 weeks with a HFD and the following 6 weeks with the HFD supplemented with the different products. For the HFD + R and HFD + W groups (Groups 3 and 4), HFD pellets were crushed in a mill along with the freeze-dried apple flesh. For HFD + A (Group 5), the *Aronia* extract was dissolved daily in the drinking water. Rats from the HFD + Atorv group (Group 6) were given the drug atorvastatin (Pfizer-Egypt Company, Cairo, Egypt) at a dosage of 4 mg/kg/day dissolved daily in the drinking water. The atorvastatin dose was adjusted if necessary according to the volume consumed and the animal’s weight. Moreover, diets of the HFD (Group 2) and Groups 5 and 6 were modified by adding 25% of chow diet in the same proportion as the apples so that all groups except Group 1 took the same proportion of HFD during the supplementation period. (Figure S1). HFD + R and HFD + A were supplemented with the same dose and type of ACNs, 1.8 and 1.9 mg/day/rat, respectively. The ACN-administered dose results in a human equivalent dose of 70 mg/day, which was calculated according to Reagan-Shaw et al. [18]. The nutritional composition regarding macronutrients and energy of each diet used in the study is shown in Supplementary Table S2. Equalization of the HFD amount was to provide similar amounts of Kcal and macronutrients (protein, carbohydrate and fat) in 5 treated groups, thus, no significant differences were observed. Supplementary Table S3 show biochemical parameters of rat plasma from the different groups studied. The table show that the group with HFD significantly increases the levels of total cholesterol, non-HDL cholesterol, alanine aminotransferase, aspartate aminotransferase and insulin compared to the group following STD diet.

During the study, rats were housed in cages on a 12 h light-12 h dark schedule at a controlled temperature (20 ± 2 °C) and humidity (55 ± 10%). Males and females were housed separately, and a maximum of two animals were housed in the same cage. For every treatment group (*n* = 6), a total of 4 cages were used: Females (*n* = 3): *n* = 2/cage and *n* = 1/cage; Males (*n* = 3): *n* = 2/cage and *n* = 1/cage. Food and water were available ad libitum. Body weight, food, and water intake were recorded every 3 days and regardless the type of diet no significant differences were observed (Supplementary Table S4).

At the end of the study, the rats were anaesthetized with isoflurane (IsoFlo, Veterinarian Esteve, Bologna, Italy) and sacrificed by cardiac puncture. The rats were perfused with an isotonic (0.9%) sodium chloride solution to remove the remaining blood in the tissues. The hearts and the descending portion of the aortas were excised and immediately snap-frozen in liquid nitrogen. All animal experiments were conducted following the European Communities Directive 2010/63/EU regulating animal research guidelines. All protocols were approved by the Animal Ethical Committee of the University of Lleida (CEEA 01-10/17) and performed under a Generalitat de Catalunya Project Licence (10038). The study complies with the ARRIVE guidelines developed by the NC3Rs [19,20].

### 2.3. Proteomic Analysis

#### 2.3.1. Protein Extraction and Quantification

To determine the total protein content, the aortas and hearts were weighed (25–30 mg) and lysed following the radioimmunoprecipitation assay buffer protocol (Thermo Fisher Scientific, Madrid, Spain) and processed as explained in more detail in Appendix A.

#### 2.3.2. Protein Digestion and Peptide 10-Plex Tandem Mass Tag (TMT) Labelling

A total of 30 μg of protein obtained from the aorta or heart tissues was reduced with 4 mM 1.4-dithiothreitol for 1 h at 37 °C and alkylated with 8 mM iodoacetamide for 30 min at 25 °C in the dark. Afterward, the samples were digested overnight (pH 8.0, 37 °C) with sequencing-grade trypsin (Promega Biotech Iberica SL, Alcobendas, Madrid, Spain) at an enzyme:protein ratio of 1:50. Digestion was quenched by acidification with 1% (*v*/*v*) formic acid, and peptides were desalted on an Oasis HLB SPE column (Waters, Cerdanyola del Vallès, Spain) before tandem mass tag (TMT) 10-plex labelling (Thermo Fisher Scientific) following the manufacturer’s instructions. Samples were normalized along with the different TMT-multiplexed batches using a TMT-126 tag labelled pool containing all the samples included in each TMT batch. The different TMT 10-plex batches were desalted on Oasis HLB SPE columns before nanoliquid chromatography coupled to mass spectrometry (LC-MS) analysis.

#### 2.3.3. Off-Gel Nano LC-(Orbitrap) MS/MS Analysis

The multiplexed and labelled aortas or hearts were fractionated by Off-gel electrophoresis (Agilent, Madrid, Spain) as instructed in the manufacturer’s protocol. Samples were fractioned into 12 nonlinear pH 3–10 fractions (Appendix A). Chromatographic separation was performed with a 90 min gradient using Milli-Q water (0.1% formic acid) and acetonitrile (0.1% formic acid) as a mobile phase at a flow rate of 300 nL/min. Mass spectrometry analyses were performed on an LTQ-Orbitrap Velos Pro from Thermo Fisher Scientific by an enhanced Fourier transform-resolution MS spectrum (R = 30,000 FHMW) followed by a data-dependent Fourier transform coupled to double mass spectrometry (FT-MS/MS) acquisition (R = 15,000 FHMW, 40% HCD) from the ten most intense parent ions with a charge state rejection of one and dynamic exclusion of 0.5 min.

#### 2.3.4. Protein Identification/Quantification

Aortic or heart protein identification and quantification were performed with Proteome Discoverer software v.1.4.0.288 (Thermo Fisher Scientific) by Multidimensional Protein Identification Technology combining the six raw data files obtained after strong cation-exchange chromatography fractionation. For protein identification, all MS and MS/MS spectra were analysed using the Mascot search engine (v.2.5) (London, UK). Mascot was set up to search the SwissProt_2018_03.fasta database (557,012 entries), restricting human taxonomy (20,317 sequences) and assuming trypsin digestion. Two missed cleavages were allowed, and an error of 0.02 Da for FT-MS/MS fragmentation mass and 10.0 ppm for an FT-MS parent ion mass were allowed. TMT-10plex was set as the quantification, modification, and oxidation of methionine, and the acetylation of N-termini was set as a dynamic modification, whereas carbamidomethylation of cysteine was set as a static modification. The false discovery rate and protein probabilities were calculated by Perclorator.

For protein quantification, the ratios between each TMT label and each 126-TMT label were used, and the results were normalized based on protein median values. Results were not confirmed by western blot because of the argument of Aebersold R. et al., [21] who confirmed that the results obtained by MS-based proteomics (also recognized by the journal *Nature Methods* as the Method of the Year 2012 [22] are vastly superior to the results obtained by Western blot for several reasons [21]. Moreover, analysing individual biological aorta or heart replicates instead of pooling samples, as is the case in our study, gives more statistical power to the differentially expressed proteins and makes the use of additional methods for validating the findings unnecessary. The MS proteomics data have been submitted to the ProteomeXchange Consortium through the PRIDE [23] partner repository with the dataset identifier PXD018885.

### 2.4. Statistical Analysis

#### 2.4.1. Data Pre-Processing

For statistical analyses, only the proteins present in ≥67% of the samples in all groups were considered. In addition, log base 2 (log2) transformations were applied to the data, including variance stabilization, data range compression, and normalization of the data distribution.

Another important advantage of using log2 transformation is the ratio comparisons, such as the fold-change (FC), when comparing, for example, the HFD vs. the SCD (HFD/SCD ratio).

Finally, the protein data set was mean-centred and Pareto scaled by being divided by the square root of the standard deviation (SD) of each variable to reduce the influence of intense peaks while emphasizing weaker peaks that may have had more biological relevance, although without giving too much relevance to noise signals.

#### 2.4.2. Multivariate Statistical Analysis

A multivariate statistical approach was initially performed on proteins identified using Metaboanalyst 4.0 (http://www.metaboanalyst.ca/, accessed on 10 January 2022) software (5 June 2018). The modelling made use of hierarchical clustering and other supervised methods, including partial least squares discriminant analysis and orthogonal projection to latent structures discriminant analysis (Appendix A). All these methods were applied using Pareto scaling.

Multivariate analysis was based on the eigendecomposition of a cross-product matrix (e.g., covariance matrix) and thus required complete datasets. To estimate missing values, we used a Bayesian principal component analysis for values missing at random. The protein component analysis was calculated using Bayes theorem, while Bayesian estimation was used to calculate the likelihood of an estimated value.

#### 2.4.3. Univariate Statistical Analysis

A univariate test was performed for each protein. For the univariate case, data were not Pareto scaled. A Kolmogorov-Smirnov test was carried out to check for distribution normality. For pairwise comparisons, either Student’s *t*-test or a Wilcoxon test was performed depending on each protein distribution. In the first case, a test for equality of variances was performed before the *t*-test analysis. *p* values were adjusted using the Benjamini-Hochberg method for multiple testing considering a 5% false discovery rate. The reported results included the fold change and the *p* values for each group. A *p*-value < 0.02 was considered to be statistically significant.

### 2.5. Clustering and Pathway Analysis

An initial functional evaluation was performed using the UniProt (www.uniprot.org, accessed on 10 January 2022) database, with a focus on protein function and relevant biological processes. Ingenuity pathway analysis (IPA software; Ingenuity System Inc., Redwood, CA, USA; www.ingenuity.com, accessed on 10 January 2022) was employed to examine the functional correlations within groups. Datasets containing protein identifiers (UniProt-KB) and their corresponding expression values (FC) of each two comparative groups were uploaded. Each protein identifier was mapped to its corresponding protein object in the Ingenuity Pathways Knowledge Base. All mapped proteins were differentially expressed with *p* < 0.05 and overlaid onto global molecular networks developed from information contained in the knowledge base. The networks were then algorithmically generated based on their connectivity. Networks were “named” in the most prevalent functional group(s) present. Networks were ranked by a score that defines the probability of a collection of nodes being equal to or greater than the number in a network achieved by chance alone. Canonical pathways, diseases, and biofunctions, ingenuity tox list, and molecular activity predictor tools were overlaid on the networks.

## 3. Results

### 3.1. Proteomic Analysis in Aorta and Heart Rat Tissues

After proteomic analysis, a total of 1163 proteins in the aorta and 1149 proteins in heart tissues from Wistar rats were identified. Complete information about relative protein quantification and identification, protein coverage and the peptides identified from the proteomic analysis in the aortas and hearts of rats is shown in Supplementary Tables S5 and S6, respectively. After the 70% frequency filter was applied, 750 proteins and 761 proteins were considered for further statistical analysis in the aorta and heart samples, respectively.

### 3.2. Tissue Proteome Modulation by Different Diets in Aorta and Heart Tissues

When we compared between groups split by sex, no differences were found in the proteomic analyses. Therefore, the results comparing the different diet groups were not split by sex (*n* = 6/group).

#### 3.2.1. HFD versus SCD

When compared to SCD treatment, HFD treatment resulted in significant increases or decreases of certain proteins in aorta and heart tissues of Wistar rats, which are described in Supplementary Table S7.

#### 3.2.2. Effects of the Red-Fleshed Apple Variety

Table 1 shows the results of the significantly up- or downregulated proteins expressed in the aorta or heart tissues after HFD + R treatment compared to the results after HFD treatment (*p* < 0.02).

#### 3.2.3. Effects of the White-Fleshed Apple Variety

The differentially expressed proteins modified after HFD + W treatment and classified by tissues are shown in Table 2. When comparing HFD + W treatment to HFD treatment, there was a decrease of seven differentially expressed proteins in the aorta and heart tissues: C3, CP, TF, SERPINA3N, C9, HP, and HPX (Figure S2; *p* < 0.02).

#### 3.2.4. Effects of Aronia Extract Infusion

After evaluating the impact of ACN diet supplementation through the apple, we evaluated the impact of ACNs and minimized the apple matrix effect. Regarding the up- or downregulation of the differentially expressed proteins modified by HFD + A treatment compared to HFD treatment, we observed that HFD + A treatment only significantly modified three proteins in heart tissue (PHYH, GLRX3, and MRPL38; *p* < 0.02)). However, in aorta tissue, many more proteins were modulated by HFD + A treatment than by HFD treatment, as shown in Table 3 (*p* < 0.02).

#### 3.2.5. HFD + Atorv versus HFD

The results of HFD + Atorv treatment are shown in Supplementary Table S8. In aortic tissue, HFD + Atorv treatment increased MYPOP and decreased SEPT9, MAP4, and FHL1 (*p* < 0.02) compared to HFD treatment alone. However, in heart tissue, HFD + Atorv treatment increased TNS1, PCBP2, DPYSL2, LMNA, GPX1, and ES1 protein homologue, mitochondrial and decreased ECH1, MB, GSTM2, PHB, CBR1, NDUFB7, PGAM1, NME2, AKR1C15, and CKMT2 (*p* < 0.02) compared to HFD treatment alone. After heart and aorta proteome analysis, the pleiotropic effects of atorvastatin, which can affect the cardiovascular system beyond affecting the lipid profile, were demonstrated [24].

#### 3.2.6. Comparisons of Red- and White-Fleshed Apple Varieties and Aronia Extract Infusion with Atorvastatin

The atorvastatin diet was used as a control due to its hypolipidemic and antioxidant properties, but it could also have other attributed effects.

**Figure 1:**
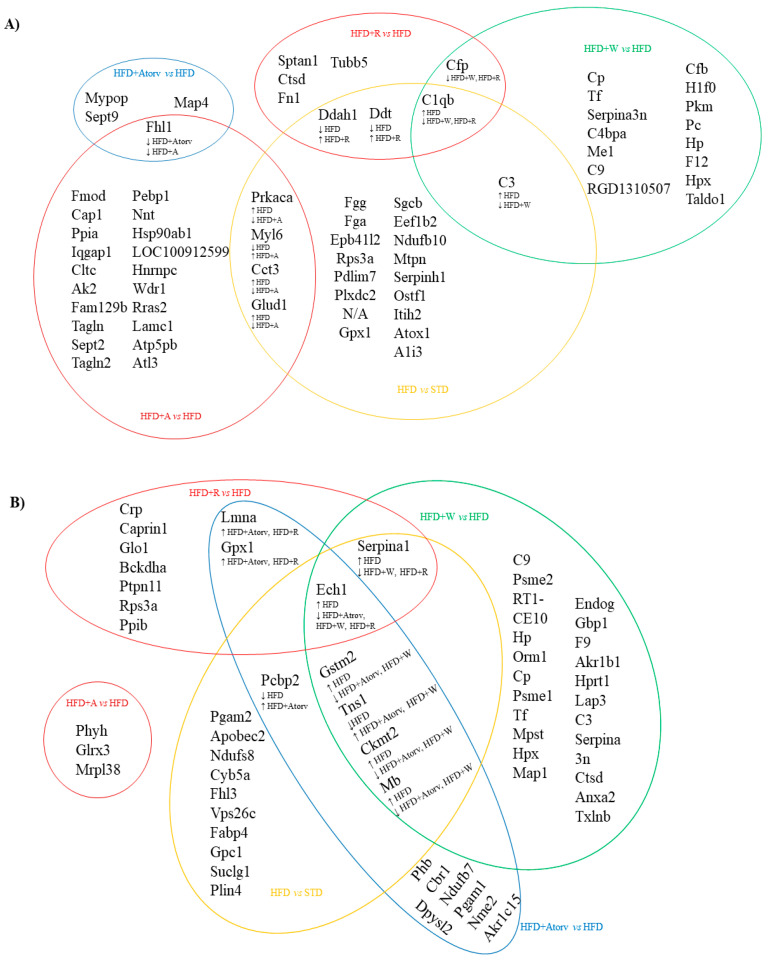
A Venn diagram is shown of the common differentially expressed proteins compared across the different treatments. (**A**) Aorta, and (**B**) heart tissue. SCD, standard chow diet; HFD, high-fat diet; HFD + R, HFD + red-fleshed apple; HFD + W, HFD + white-fleshed apple; HFD + A, HFD + Aronia (anthocyanin-rich extract); HFD + Atorv, HFD + Atorvastatin. The common proteins up- (↑) or downregulated (↓) by the different treatments are shown in the diagram.

In addition to the apple matrix effect, it was noted that rats treated with HFD + W, HFD + R, or HFD + A exhibited a change in proteins that were also modified by HFD + Atorv intervention (Figure 1), which was the positive control. HFD + Atorv treatment decreased the expression of ECH1, which was also observed in rats after HFD + W and HFD + R interventions, an effect that has not been reported to date by other authors.

In addition, GPX1 was downregulated in the HFD + R group, while GSTM2 and MB were both downregulated in the HFD + Atorv and HFD + W groups. Finally, both the HFD + A and the HFD + Atorv groups showed the same downregulation in the four-and-a-half LIM domains protein 1 (FHL1), a protein with unknown function. However, FHL1 is significantly increased in cardiac failure, cardiac hypertrophy, pulmonary hypertension, and arrhythmias [25]. Therefore, we suggest that apples and atorvastatin share common mechanisms of action that positively impact diverse CVD risk factors.

A summary of the main findings after the HFD + R, HFD + W, and HFD + A treatments is presented in Supplementary Figure S3.

### 3.3. Common Proteins Modified by the Different Diets in the Aorta and Heart Tissue

#### 3.3.1. Aorta Tissue

The common differentially expressed proteins in the aortic tissue after the different treatments are represented in Figure 1A.

FHL1 was reduced in aorta tissue after HFD + Atorv (−1.465 FC, *p* = 0.0108) and HFD + A (−1.669 FC, *p* = 0.0022) treatments compared to the FHL1 level in the HFD group.

PRKACA (−1.122 FC, *p* = 0.0043), CCT3 (−1.634 FC, *p* = 0.0152), and GLUD1 (−1.256 FC, *p* = 0.0162) were reduced, and MYL6 (1.653 FC, *p* = 0.0119) was increased after HFD + A treatment compared to HFD treatment. However, the inverse effect was observed after HFD treatment as PRKACA, CCT3, and GLUD1 expression increased (1.240 FC; *p* = 0.0064, 1.622 FC; *p* = 0.0087, and 1.297 FC; *p* = 0.0118, respectively) and MYL6 expression decreased (−1.631 FC, *p* = 0.0167) compared to expression in the SCD group.

DDAH1 (1.554 FC, *p* = 0.0090) and DDT (1.427 FC, *p* = 0.0164) expression increased after HFD + R treatment compared to expression in the HFD group, and the same proteins were downregulated after HFD (−1.365 FC, *p* = 0.0190 and −1.466 FC, *p* = 0.0120, respectively) treatment when compared to SCD treatment.

C3 expression (−2.158 FC, *p* < 0.0001) decreased after HFD + W treatment when compared to expression after HFD treatment, but C3 expression was increased after HFD treatment (1.489 FC, *p* = 0.0019) when compared to expression after SCD treatment.

C1QB expression decreased after HFD + W (−2.554 FC, *p* = 0.0004) and HFD + R (−1.674 FC, *p* = 0.0087) treatments when compared to expression after HFD treatment, and C1QB expression increased after HFD treatment (2.001 FC, *p* = 0.0111) when compared to expression after SCD treatment.

CFP expression decreased after HFD + W (−1.701 FC, *p* = 0.0087) and HFD + R (−1.454 FC, *p* = 0.0152) treatments when compared to expression after HFD treatment.

Thus, in rat aorta tissue, CFP and C1QB decreased after HFD + W and HFD + R treatments, suggesting an apple matrix effect. DDAH1 and DDT were increased after HFD + R treatment, suggesting an ACN effect provided by the red-fleshed apple variety. PRKACA, CCT3, FHL1, and GLUD1 were also reduced in the aorta, while MYL6 was increased after HFD + A treatment, suggesting an Aronia extract infusion effect.

#### 3.3.2. Heart Tissue

The common differentially expressed proteins in heart tissue that were modified after different treatments are represented in Figure 1B.

In heart tissue, HFD + A treatment did not modify any of the proteins modified by the other treatments.

PCBP2 expression increased after HFD + Atorv treatment (1.245 FC, *p* = 0.0022) when compared to that of HFD treatment, and PCBP2 expression decreased after HFD treatment (−1.273 FC, *p* = 0.0022) when compared to that of STD treatment.

GSTM2 (−1.354 FC, *p* = 0.0041), CKMT2 (−1.499 FC, *p* = 0.0172), and MB (−1.727 FC, *p* = 0.0027) expression levels were reduced after HFD + Atorv and HFD + W treatments (−1.326 FC; *p* = 0.0047, −1.418 FC; *p* = 0.0116, and −1.512 FC; *p* = 0.0199, respectively) when compared to the levels after HFD treatment; however, GSTM2 (1.386 FC, *p* = 0.0034), CKMT2 (1.532 FC, *p* = 0.0015), and MB (1.609 FC, *p* = 0.0100) expression levels were increased after HFD treatment when compared to the levels after SCD treatment.

However, the TNS1 level was increased after HFD + Atorv (1.297 FC, *p* = 0.0008) and HFD + W (1.273 FC, *p* = 0.0083) treatments compared to the level after HFD treatment, but the TNS1 level was decreased after HFD (−1.280 FC, *p* = 0.0176) treatment compared to that after SCD treatment.

LMNA and GPX1 expression levels were increased after HFD + Atorv (1.267 FC, *p* = 0.0023 and 1.177 FC, *p* = 0.0065, respectively) and HFD + R (1.153 FC, *p* = 0.0187 and 1.179 FC, *p* = 0.0110, respectively) treatments when compared to the levels in the HFD group.

SERPINA1 expression was decreased after HFD + W (−1.273 FC, *p* = 0.0188) and HFD + R (−1.263 FC, *p* = 0.0054) treatments when compared to expression in the HFD group. In contrast, SERPINA1 expression increased after HFD treatment (1.234 FC, *p* = 0.0177) when compared to expression in the SCD group. ECH1 expression was decreased after HFD + Atorv (−1.298 FC, *p* = 0.0012), HFD + W (−1.305 FC, *p* = 0.0152), and HFD + R (−1.144 FC, *p* = 0.0133) treatments when compared to expression in the HFD group. However, ECH1 expression increased after HFD treatment (1.175 FC, *p* = 0.0083) when compared to expression in the SCD group.

In rat heart tissue, GSTM2, CKMT2, and MB expression levels were reduced and TNS1 expression was increased after HFD + W treatment, suggesting a differential effect of the white-fleshed apple variety. SERPINA1 and ECHI were decreased after HFD + W and HFD + R treatments, suggesting an apple matrix effect. LMNA and GPX1 expression levels were increased after HFD + Atorv and HFD + R treatments, suggesting an ACN effect induced by the red-fleshed apple variety that is similar to the hypolipidemic drug’s effect.

### 3.4. Pathway Analysis of the Differentially Expressed Proteins in Heart and Aorta Tissues Modulated by HFD + W or HFD + A Treatments

To evaluate the effects of anthocyanins in apple flesh, we conducted clustering and pathway analysis using IPA software with the differentially expressed proteins (*p* < 0.02) in Wistar rat aorta and heart tissues after HFD + W treatment, as a standard apple intake source, or HFD + A treatment, as an anthocyanin-apple source. Due to the origin of the differentially expressed proteins modified by HFD + R treatment, pathway analysis could not be predicted by IPA software to construct a predicted network.

**Figure 2:**
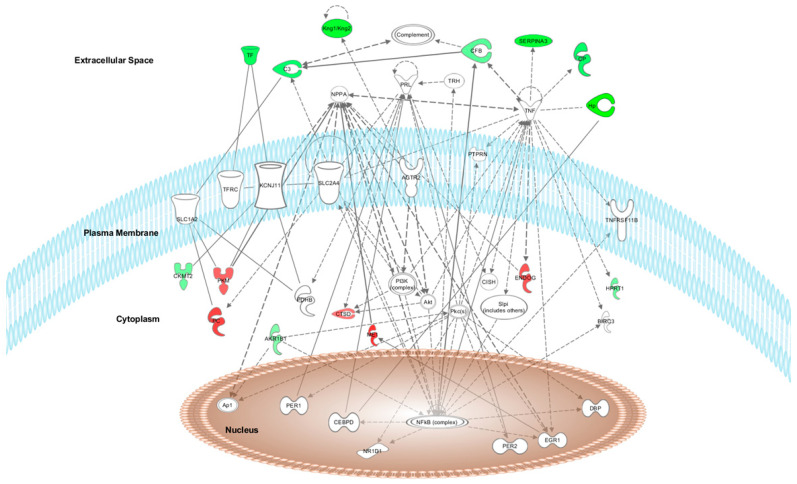
Network of neurological, haematological and cardiovascular disease systems showing the differentially expressed proteins in Wistar rat heart and aorta tissues after HFD + white-fleshed apple treatment. Proteins are represented in red or green if the proteins are up- or downregulated, respectively.

After HFD + W treatment, the top network found by IPA software was “Neurological Disease, Haematological Disease, and Cardiovascular Disease” (score = 25). Fifteen of the 46 differentially expressed proteins formed part of this network (Figure 2).

**Figure 3:**
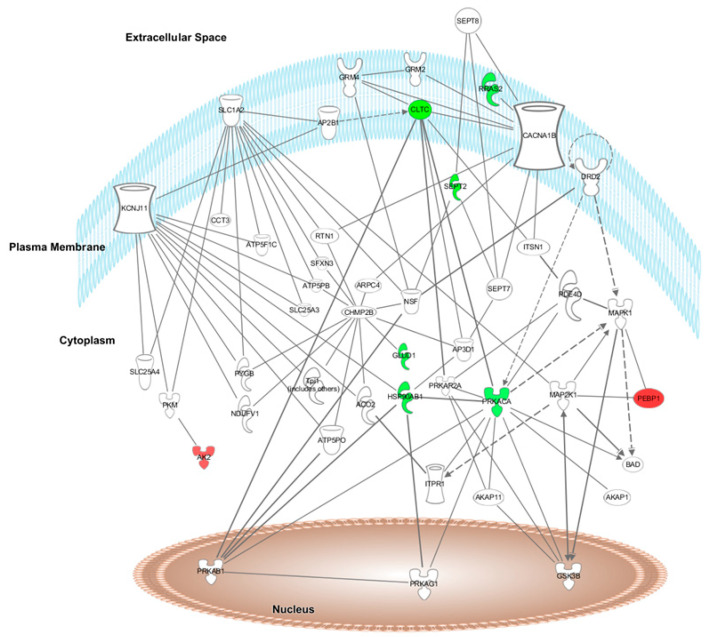
Network of energy production, cellular function and maintenance, and posttranslational modifications showing the differentially expressed proteins in Wistar rat heart and aorta tissues after HFD + Aronia (anthocyanin-rich extract) treatment. Proteins are represented in red or green if the proteins are up- or downregulated, respectively.

Furthermore, after HFD + A treatment, the top network found by IPA software was “Energy Production, Cellular Function and Maintenance, and Post-translational Modifications” (score = 17). Ten of the 28 differentially expressed proteins formed part of this network (Figure 3).

The graphical representation of the main networks modified by HFD + W or HFD + A treatments is shown in Figure 2 and Figure 3, respectively, in which the modulated proteins or other predicted proteins involved in the network are located in the cell compartments The differentially expressed proteins modified by HFD + W or HFD + A treatment are highlighted in colour and indicate when the protein expression is up- or downregulated (red means upregulated and green means downregulated) when compared to expression in the HFD group.

The top canonical pathways modulated after proteome analysis of HFD + W treatment were acute phase response signalling (C3, C9, C4BPA, CFB, CP, HP, HPX, ORM1, SERPINA1, SERPINA3, and TF), complement system (C3, C9, C1QB, C4BPA, and CFB), LXR/RXR activation (C3, C9, HPX, ORM1, SERPINA1, and TF), FXR/RXR activation (C3, C9, HPX, ORM1, SERPINA1, and TF) and the coagulation system (F9, F12, and SERPINA1) iron homeostasis signalling pathway (CP, HP, HPX, and TF). The top canonical pathways modulated after proteome analysis of HFD + A treatment were CDK5 signalling (LAMC1, PRKACA, and RRAS2), epithelial adherens junction signalling (IQGAP1, MYL6, and RRAS2), glutamate biosynthesis II (GLUD1), glutamate degradation X (GLUD1), PPARα/RXRα activation (HSP90AB1, PRKACA, and RRAS2), and actin cytoskeleton signalling (IQGAP1, MYL6, and RRAS2).

### 3.5. Upstream Regulators of the Protein Dataset Modified after HFD + W or HFD + A Treatment of Heart and Aorta Rat Tissues

The IPA software identified the cascade of upstream transcriptional regulators that can explain the observed gene expression changes in the protein dataset. After HFD + W treatment, the top five upstream regulators of the protein dataset modified in aorta and heart rat tissues were TNF, AGTR2, NFE2L2, EGR1, and PRL. After HFD + A treatment, the top five upstream regulators of the protein dataset modified in aorta and heart Wistar rat tissues were FLNA, FRS2, MYOCD, PIAS1, and Yap1. These regulators help to illuminate the biological activities occurring in tissues or cells.

### 3.6. Top Relevant Diseases and Biological Functions Affected by HFD + W or HFD + A Treatment

The top relevant diseases and biological functions affected by HFD + W treatment in aorta and heart tissues included the following: blood coagulation (C3, C9, F12, and F9), homeostasis of iron (CP and TF), complement activation (C3 and CFB), transport of transition metal ions (CP and TF), complement-mediated lysis of red blood cells (CFB), classical complement pathway (C3), transport of iron ion (TF), transport of Cu2+ (CP), myocardial infarction (C3), and contraction of aortic ring tissue (HPX).

The top relevant diseases and biological functions affected by HFD + A treatment were nervous system development (PRKACA, CLTC, CAP1, SEPT2, and IQGAP1), cell death and survival (PRKACA, RRAS2, HSP90AB1, PPIA, and NNT) and small molecule biochemistry (AK2, ATP5PB, GLUD1, and NNT).

## 4. Discussion

The main objective of this study is describing the proteomic profile of the rat heart and aorta tissues after the sustained intake of a high-fat diet supplemented with a white-fleshed apple (anthocyanin-poor), a red-fleshed apple (anthocyanin-rich), or with an anthocyanin-rich infusion, in order to determine their effects against a HFD. Moreover, our study, for the first time, provides insights on how apples alter the expression of different CVD related proteins in the rat heart and aorta tissues by a proteome profile analysis. In the frame of this study, our previous work showed cardiometabolic protective effects of both red-fleshed and white-fleshed apples and also aronia infusion supplementation, specifically in the significant reduction of the aorta thickness [26]. Although no gender differences were observed in this proteomics study, we observed gender differences in some cardiometabolic parameters [26]. Specifically, we observed that the kidney function was improved after all supplementations (both apples and aronia) but only in females (probably related to the higher phenol bioavailability reported in females). We also observed only in males a decrease in insulin plasma concentration after ingestion of both apples.

### 4.1. Effects of the Red-Fleshed Apple Variety on Cardiovascular-Related Proteins

In aorta tissues, HFD + R treatment significantly (*p* < 0.02) upregulated the expression of N(G),N(G)-dimethylarginine dimethylaminohydrolase 1 (DDAH1) compared to HFD treatment. DDAH1 is an enzyme that catalyses the hydrolysation of two endogenous inhibitors of NO synthases, inhibiting their protective activity against cardiovascular morbidity [27]. Significantly upregulated expression of both DDAH1 and DDAH2 was induced by ACNs isolated from cornelian cherry fruit, which was introduced by diet to atherosclerotic New Zealand rabbits [28].

Additionally, in the present study, HFD + R treatment significantly upregulated the expression of glutathione peroxidase 1 (GPX1) in heart tissue. GPX1 is an antioxidant enzyme that can restore an endothelial phenotype in some types of pathology with high levels of oxidative stress, such as hyperhomocysteinemia [29], and the activity of GPX1 has been inversely correlated with CVD in patients with coronary artery disease [30].

In the hearts of Wistar rats, our findings demonstrate for the first time that the ingestion of a HFD supplemented with ACN-rich apples can significantly upregulate GPX1 expression despite the detrimental effects of an HFD [28].

In rat aorta tissues, HFD + R supplementation significantly upregulated the expression of cathepsin D (Table 1), a cholesterol efflux-inducing molecule that increases the expression of phospholipid-transporting ATPase 1 (ABCA1) and apolipoprotein A-I and mediates lipid efflux [31].

Therefore, HFD + R mediated the upregulation of DDAH1 in the aorta and GPX1 in heart tissue, consistent with a healthier pattern of CVD biomarkers in rats. These findings support the beneficial role of red-fleshed apples rich in ACNs for the prevention of CVD.

### 4.2. Effects of the Red-Fleshed Apple Variety on CRP, Complement System Proteins, and Energy Homeostasis

In heart tissue, HFD + R treatment significantly reduced the expression of CRP compared to HFD treatment, suggesting an anti-inflammatory effect (Table 1). CRP is a proinflammatory molecule involved in diverse reactions that are related to the activation of the inflammatory process [32] associated with the development of atherosclerosis and other cardiovascular events [33].

In addition, in aorta tissue, HFD + R treatment significantly downregulated the expression of C1QB and CFP involved in the complement system (Table 1) and downregulated ECH1 involved in energy homeostasis in heart tissue. As discussed below, similar changes were observed in the HFD plus white-fleshed apple (HFD + W) group.

### 4.3. Effects of the White-Fleshed Apple Variety on the Complement System and Anti-Inflammatory Proteins

HFD + W treatment downregulated the expression of proteins involved in the activation of both the classical and alternative complement pathways, such as complement factor 3 (C3) and C9 in the hearts and aortas of rats, while complement factor B (CFB), properdin (CFP), C4BPA, and C1QB were reduced only in rat aortic tissue.

A decrease in the C3 concentration produces a reduction in the spontaneous conversion of C3 into hydrolysed C3 [C3(H_2_O)] [34]. In turn, C3 (H_2_O) should functionally bind to the CFB, which was also downregulated by HFD + W treatment, and to complement factor D (CFD) to generate the metastable molecule C3b, a key opsonizing molecule that is part of the innate immune system [34,35], protecting against infections in mammals.

HFD + W treatment also significantly downregulated the expression of C4 binding protein alpha chain (C4BPA) and complement C1q subcomponent subunit B (C1QB) in the rat aorta while reducing complement factor 9 (C9) in aorta and heart tissues (Table 2). A reduction in C9 expression could reflect a reduction in the atherosclerotic plaque formation process, since it has been demonstrated that high concentrations of C9 are present as deposits in the intima layer of grade II atherosclerotic lesions in the human aorta [36]. Hence, the novel results of the present study regarding the effects of HFD + W supplementation in rats showed significant downregulation in the expression of proteins involved in the complement system, such as CFP, CFB, C3, C4BPA, C1QB, and C9. Such downregulation might be involved in the reduction of CVD risk, as the formation of atherosclerotic plaques is a complex process performed between modified lipid particles and diverse innate immune system molecules [37]. Additionally, HFD + W supplementation in rats reduced other proinflammatory molecules, such as SERPINA 1 (α1-antitrypsin) in heart tissue and SERPINA3N (α1-antichymotrypsin), in both the heart and aorta, suggesting a positive effect of HFD + W supplementation on cardiovascular risk through the regulation of the inflammatory process.

### 4.4. Effects of the White-Fleshed Apple Variety on Iron Homeostasis Proteins

As a result of our experiments, when compared to HFD treatment, HFD + W treatment significantly reduced the expression of proteins involved in iron homeostasis, such as myoglobin (MB), in the heart tissue while reducing the expression of haptoglobin (HP), serotransferrin (TF), haemopexin (HPX), and ceruloplasmin (CP) in heart and aorta tissues (Table 2).

The iron-binding myoglobin (MB) molecule serves as a dioxygen reservoir in the muscles of mammals [38]. MB can act as a potent nitric oxide (NO) scavenger, thus representing a control system for the preservation of mitochondrial respiration [39]. These findings suggest that a reduction in the expression of myoglobin might be beneficial for hypertensive states when there is a reduced bioavailability of vascular NO [40]. Moreover, HFD + W supplementation downregulated the expression of CP, a copper-binding glycoprotein with ferroxidase activity and antioxidant properties [41], which is linked to the promotion of deleterious vascular effects that are a risk factor for CVD [42,43]. In addition to the aforementioned effects, HFD + W treatment significantly reduced the expression of transferrin, an iron-binding protein that controls ferric iron concentrations in human body fluids [44].

High transferrin concentrations (>160 mg/dL) are associated with an increased CVD mortality risk in individuals with elevated transferrin and LDLc levels [45]. Thus, HFD + W supplementation resulted in a decrease in myoglobin, transferrin, and ceruloplasmin proteins involved in iron homeostasis, which participates in essential reduction-oxidation reactions for several fundamental biological processes.

Finally, HFD + W treatment significantly reduced the expression of HP, an acute-phase protein, in heart and aortic tissues. This reduction is considered positive, and it has also been observed in a study with olive oil phenolic compounds [46], where the reduction in the expression of haptoglobin was related to an improvement in the cholesterol efflux capacity of the HDL particles in humans. Therefore, HFD + W supplementation can exert a positive effect on CVD through the regulation of iron homeostasis-related proteins.

### 4.5. Effects of the White-Fleshed Apple Variety on Energy Homeostasis Proteins

HFD + W treatment downregulated the expression of enoyl-CoA hydratase 1 (ECH1) in heart tissue (Table 2), an enzyme that catalyses the second step in fatty acid β-oxidation and the metabolization of branched-chain amino acids [47]. The downregulation of ECH1 has been linked to enhanced resistance to ischaemia-reperfusion injury in the hearts of Brown Norway rats [48].

Moreover, HFD + W supplementation also significantly downregulated the expression of glutathione S-transferase Mu 2 (GSTM2) in the heart, a molecule that reduces the activity of ryanodine receptors in the sarcoplasmic reticulum, causing a reduction in spontaneous contraction frequency and myocyte shortening [49], therefore improving heart contractility.

One interesting finding is that HFD + W treatment downregulated the expression of annexin A2 in hypercholesterolaemic heart rat tissue, a calcium-regulated binding protein that reduces the expression of the proprotein convertase subtilisin/Kexin Type 9 (PCSK9) enzyme [50]. The PCSK9 receptor is an enzyme known for its capability to bind LDL receptors (LDLRs) on the liver, promoting their degradation [51]; hence, a reduction in the degradation of LDLRs increases the clearance of cholesterol inside LDL molecules, consequently reducing LDLc plasma levels.

Moreover, HFD + W treatment upregulated the expression of pyruvate kinase in the aorta and pyruvate carboxylase and NADP-dependent malic enzyme (ME1) in heart tissue. The upregulation of these enzymes increases the intracellular concentrations of oxaloacetate and malate, substrates needed to start the tricarboxylic acid cycle, suggesting a possible increase in intracellular energy levels [52].

### 4.6. Effects of Aronia Extract Infusion on Cellular Signalling Proteins

In the aorta, *Aronia* extract infusion significantly modified the expression of different proteins, including downregulation of protein kinase cAMP-dependent catalytic alpha (PRKACA). The decrease in PRKACA expression observed after HFD + A supplementation would favour the inhibition of spontaneous and pathological blood clot formation in blood vessels [53], potentially reducing the risk of cardiovascular events.

Additionally, HFD + A treatment downregulated the expression of IQ motif containing GTPase activating protein 1 (IQGAP1), a protein with a crucial role in regulating the assembly and dynamics of the actin cytoskeleton, in aortic tissue (Table 3). IQGAP1 overexpression has also been associated with cell proliferation, migration, and rearrangement of vascular smooth muscle cells in varicose veins [54].

HFD + A treatment also downregulated the expression of the heat shock protein HSP 90-beta (HSP90AB1) in the aorta. HSP90AB1 is necessary for a large number of cellular processes, acting as a chaperone promoting the maturation and structural maintenance of different proteins involved in cell cycle control and signal transduction [55,56].

### 4.7. Effects of Aronia Extract Infusion on Cellular Structure-Related Proteins

In the aorta, HFD + A treatment upregulated fibromodulin (FMOD) in rats. FMOD protein participates in the assembly of collagen fibres in the extracellular matrix and is known to trigger platelet aggregation through the activation of a collagen-specific receptor [57]. This upregulation supports an interest in ACNs as a positive modulator of the intravascular coagulation process.

Additionally, in the aorta, HFD + A treatment increased the aortic expression of transgelin (TAGLN) and TAGLN2, proteins that are involved in the calcium-related contractile properties of the cell [58]. Moreover, HFD + A treatment upregulated the aortic protein expression of myosin light polypeptide 6 (MYL6), a structural protein that acts as a noncalcium binding regulatory protein of myosin [59].

Furthermore, in the aorta, HFD + A treatment significantly downregulated the expression of adenylyl cyclase-associated protein 1 (CAP1), a human resistin receptor that increases the expression of CD36 mRNA, associated with coronary artery disease [60]. Moreover, CAP1 has also been identified as an important regulator of PCSK9, a modulator of LDL receptor degradation in the liver [61].

### 4.8. The Apple Matrix Effect

In the aortas of hypercholesterolaemic rats treated with HFD + R or HFD + W, regardless of the ACN content, significant downregulation of CFP and C1QB was observed, indicating a matrix effect that could be attributed to other phenolic compounds present in the apples or other bioactive components, such as fibre. In this sense, the apple phenolic composition contains more than just anthocyanins, and the observed effects may be due to the apple phenolic phytocomplex that could act synergistically to beneficially impact the aorta and heart proteomes. Moreover, our group also recently observed an apple matrix effect between the red-fleshed apple variety and *Aronia* extract infusion, demonstrating a higher bioavailability and excretion of ACN after *Aronia* extract infusion supplementation [26]. The differences observed could be related to the fact that the ACNs in apples are bound to fibre, while in the *Aronia* extract infusion, the ACNs are more available in their free forms, which favours their gastrointestinal absorption and metabolism.

C1QB is a protein related to the activation of the complement classical pathway due to its important role as an important fragment of C1, which is the first component and main activator of the classical pathway of the complement system [62]. The downregulated effects of the apple matrix on C1QB were accompanied by a reduction in the complement system regulator CFP [63], thus leading to a stimulus for the reduction in complement system activation. HFD + W treatment significantly decreased the expression of CFP and C1QB compared with that in the HFD + R supplemented group.

In addition, in aortic tissue, HFD + R treatment significantly downregulated the expression of SERPINA1 and ECH1 in heart tissue; thus, HFD + R treatment showed similar effects to those observed with HFD + W treatment. Consequently, apple consumption, independent of the ACN content present in red-fleshed apples, induced a comparable effect on the aortic proteome involved in the complement system.

Furthermore, the HFD + R and HFD + W treatments downregulated the expression of ECH1 in rat hearts, showing the same effects as those observed with HFD + Atorv treatment.

Generally, the food matrix is viewed as a physical domain that contains and/or interacts with specific food constituents (nutrients, micronutrients, fibres and phytochemicals) exhibiting functionalities and behaviours that are different from those exhibited by a given isolated constituent [64]. Our results indicate that, regardless of the ACN content in the red-fleshed apple variety, other components in the food matrix may have an impact on the modulation of the proteome profile, similar to the white-fleshed apple matrix. However, the matrix effect has been poorly studied in apple. In accordance with our results, a previous study reported changes in the gene expression profiles of inflammatory stress following apple product intake compared to an apple phenolic extract, suggesting the modulation of a range of biological processes related to the apple matrix that could counteract the proinflammatory response induced by a high-fat meal [65].

## 5. Conclusions

Thus, considering all the above mentioned results, the red-fleshed apple variety, white-fleshed apple variety, and *Aronia* extract infusion were all able to modify the expression of multiple proteins in aorta and heart tissues in hypercholesterolaemic rats and altered different pathways, which are positively related to the CVD benefits. Moreover, no adverse events were reported and all products were well tolerated.

Specifically, the red-fleshed apple variety was involved in the downregulation of C1QB and CFP in aortic tissue and CRP in heart tissue, which relate to the complement system and inflammation.

White-fleshed apple consumption induced the downregulation of proteins involved in the complement system (C1QB, CFB, CFP, C9, and C3 in aortic tissue and C9 and C3 in heart tissue) and the iron homeostasis system (CP, HP, TF, and HPX in aortic tissue and HP, TF, HPX, and MB in heart tissue), while regulated proteins were positively involved in cellular energetic homeostasis (upregulation of ME1, PKM, and PC in aortic tissue and downregulation of ECH1, GSTM2, and ANXA2 in heart tissue).

Moreover, red-fleshed and white-fleshed apple consumption, independent of the ACN content, downregulated proteins involved in the complement system, suggesting an anti-inflammatory effect of the apple matrix, which could be related to phenolic compounds other than ACN or could involve other apple components, such as soluble fibre.

In parallel, *Aronia* extract infusion significantly regulated proteins involved in cellular structure (upregulation of FMOD, TAGLN, TAGLN2, and MYL6 and downregulation of CAP1) while downregulating proteins involved in cellular signalling pathways (PRKACA, IQGAP1, and HSP90AB1) in rat aortic tissue.

The proteomic data revealed more information about the metabolic pathways modulated by apple ACNs and the apple matrix, thereby increasing our understanding of the underlying mechanisms by which apples regulate protein expression to potentially protect the heart and aorta tissues from CVD.

Our results reveal that both types of apples showed anti-inflammatory effects through the complement system, while the red-fleshed apple variety showed a CRP reduction. In the aorta, *Aronia* extract infusion modified the expression of different structural and signalling proteins related to CVD. Moreover, regardless of the ACN content, the apple matrix, which involves different bioactive components, reduced the expression of inflammatory proteins in the aorta and/or heart. Therefore, these findings provide a more complete picture of the biological effects of apple intake on inflammation and other aspects of cellular biology.

## Figures and Tables

**Table 1:** Proteome changes on aorta or heart tissue of high-fat diet + red fleshed apple versus high-fat diet.

Tissue	UniProt ID	Gene	Protein Name	FC	*p*-Value *
AORTA	P24268	*Ctsd*	Cathepsin D	1.284	0.0083
	O08557	*Ddah1*	N(G),N(G)-dimethylarginine dimethylaminohydrolase 1	1.554	0.0090
	P80254	*Ddt*	D-dopachrome decarboxylase	1.427	0.0164
	Q6IRK8	*Sptan1*	Spectrin alpha chain, non-erythrocytic 1	−1.580	0.0081
	G3V7N9	*C1qb*	Complement C1q subcomponent subunit B/Adiponectin A	−1.674	0.0087
	F1LST1	*Fn1*	Fibronectin	−1.600	0.0136
	B0BNN4	*Cfp*	Complement factor properdin	−1.454	0.0152
	P69897	*Tubb5*	Tubulin beta-5 chain	−1.202	0.0161
HEART	M0RAM5	*Gpx1*	Glutathione peroxidase	1.179	0.0110
	P41499	*Ptpn11*	Tyrosine-protein phosphatase non-receptor type 11	1.340	0.0165
	P49242	*Rps3a*	40S ribosomal protein S3a	1.153	0.0180
	P24368	*Ppib*	Peptidyl-prolyl cis-trans isomerase B	1.342	0.0185
	G3V8L3	*Lmna*	Lamin A, isoform CRA_b	1.153	0.0187
	P48199	*Crp*	C-reactive protein	−1.653	0.0031
	Q5M9G3	*Caprin1*	Caprin-1	−1.507	0.0036
	A0A0G2JY31	*Serpina1*	Alpha-1-antiproteinase	−1.263	0.0054
	Q6P7Q4	*Glo1*	Lactoylglutathione lyase	−1.338	0.0121
	Q62651	*Ech1*	Delta(3,5)-Delta(2,4)-dienoyl-CoA isomerase, mitochondrial	−1.144	0.0133
	P11960	*Bckdha*	2-oxoisovalerate dehydrogenase subunit alpha, mitochondrial (Fragment)	−1.212	0.0152

FC, fold change. *t*-test or Wilcoxon test of pairwise comparisons was performed depending on each protein’s distribution. * *p* < 0.02 was considered statistically significant.

**Table 2:** Proteome changes on aorta or heart tissue of high-fat diet + white fleshed apple versus high-fat diet.

Tissue	UniProt ID	Gene	Protein Name	FC	*p*-Value *
AORTA	A0A0G2K1S6	*Me1*	Malic enzyme	1.670	0.0022
	P43278	*H1f0*	Histone H1.0	1.259	0.0110
	P11980	*Pkm*	Pyruvate kinase PKM	1.268	0.0128
	P52873	*Pc*	Pyruvate carboxylase, mitochondrial	1.538	0.0140
	Q9EQS0	*Taldo1*	Transaldolase	1.293	0.0187
	M0RBF1	*C3*	Complement C3	−2.158	<0.0001
	G3V7K3	*Cp*	Ceruloplasmin	−2.244	<0.0001
	G3V7N9	*C1qb*	Complement C1q subcomponent subunit B	−2.554	0.0004
	P12346	*Tf*	Serotransferrin	−2.387	0.0007
	A0A0H2UHI5	*Serpina3n*	Ab1-233	−2.793	0.0013
	Q63514	*C4bpa*	C4b-binding protein alpha chain	−2.706	0.0017
	Q62930	*C9*	Complement component C9	−3.006	0.0022
	A0A0G2K896	*RGD1310507*	Similar to RIKEN cDNA 1300017J02	−1.780	0.0038
	B0BNN4	*Cfp*	Complement factor properdin	−1.701	0.0087
	G3V615	*Cfb*	Complement factor B	−1.526	0.0094
	A0A0H2UHM3	*Hp*	Haptoglobin	−2.912	0.0152
	D3ZTE0	*F12*	Coagulation factor XII	−1.323	0.0158
	P20059	*Hpx*	Hemopexin	−1.925	0.0167
HEART	F1LN42	*Tns1*	Tensin 1	1.273	0.0083
	Q3V5X8	*Endog*	Endonuclease G	1.376	0.0120
	P24268	*Ctsd*	Cathepsin D O	1.131	0.0168
	A0A0G2K2T1	*Txlnb*	Taxilin beta	1.239	0.0186
	Q62930	*C9*	Complement component C9	−2.267	<0.0001
	Q63798	*Psme2*	Proteasome activator complex subunit 2	−1.997	<0.0001
	Q6MG34	*RT1-CE10*	RT1 class I, CE10	−2.250	<0.0001
	A0A0H2UHM3	*Hp*	Haptoglobin	−2.728	0.0006
	P02764	*Orm1*	Alpha-1-acid glycoprotein	−3.506	0.0007
	G3V7K3	*Cp*	Ceruloplasmin	−2.038	0.0016
	Q6P9V7	*Psme1*	Proteasome (Prosome, macropain) activator subunit 1	−2.216	0.0043
	P12346	*Tf*	Serotransferrin	−1.928	0.0043
	P97532	*Mpst*	3-mercaptopyruvate sulfurtransferase	1.205	0.0047
	P08010	*Gstm2*	Glutathione S-transferase Mu 2	−1.326	0.0047
	P20059	*Hpx*	Hemopexin	−1.891	0.0064
	P01048	*Map1*	T-kininogen 1	−2.852	0.0092
	P09605	*Ckmt2*	Creatine kinase S-type, mitochondrial	−1.418	0.0116
	D3ZKU6	*Gbp1*	Uncharacterized protein	−1.603	0.0123
	P16296	*F9*	Coagulation factor IX	−1.183	0.0129
	P07943	*Akr1b1*	Aldose reductase	−1.244	0.0134
	P27605	*Hprt1*	Hypoxanthine-guanine phosphoribosyltransferase	−1.446	0.0137
	Q68FS4	*Lap3*	Cytosol aminopeptidase	−1.443	0.0151
	Q62651	*Ech1*	Delta(3,5)-Delta(2,4)-dienoyl-CoA isomerase, mitochondrial	−1.305	0.0152
	M0RBJ7	*C3*	Complement C3	−1.563	0.0153
	A0A0H2UHI5	*Serpina3n*	Ab1-233	−1.809	0.0161
	Q07936	*Anxa2*	Annexin A2	−1.182	0.0176
	A0A0G2JY31	*Serpina1*	Alpha-1-antiproteinase	−1.273	0.0188
	Q9QZ76	*Mb*	Myoglobin	−1.512	0.0199

FC, fold change. *t*-test or Wilcoxon test of pairwise comparisons was performed depending on each protein’s distribution. * *p* < 0.02 was considered statistically significant.

**Table 3:** Proteome changes on aorta or heart tissue of high-fat diet + Aronia extract infusion versus high-fat diet.

Tissue	UniProt ID	Gene	Protein Name	FC	*p*-Value *
AORTA	G3V6E7	*Fmod*	Fibromodulin	1.495	0.0002
	P10111	*Ppia*	Peptidyl-prolyl cis-trans isomerase A	1.290	0.0049
	A0A0G2JSG6	*Ak2*	Adenylate kinase 2, mitochondrial	1.296	0.007
	P31232	*Tagln*	Transgelin	1.379	0.0099
	A0A0G2K6J5	*Myl6*	Myosin light polypeptide 6	1.653	0.0119
	Q5XFX0	*Tagln2*	Transgelin-2	1.360	0.0123
	P31044	*Pebp1*	Phosphatidylethanolamine-binding protein 1	1.569	0.0129
	D3ZCZ9	*LOC100912599*	NADH dehydrogenase [ubiquinone] iron-sulfur protein 6, mitochondrial	1.492	0.0139
	Q9WUH4	*Fhl1*	Four and a half LIM domains protein 1	−1.669	0.0022
	Q08163	*Cap1*	Adenylyl cyclase-associated protein 1	−1.472	0.0037
	A1L1M0	*Prkaca*	Protein kinase, cAMP-dependent, catalytic, alpha	−1.122	0.0043
	G3V7Q7	*Iqgap1*	IQ motif containing GTPase activating protein 1 (Predicted), isoform CRA_b	−1.507	0.006
	F1M779	*Cltc*	Clathrin heavy chain	−1.582	0.0064
	B4F7E8	*Fam129b*	Niban-like protein 1	−1.204	0.0091
	Q91Y81	*Sept2*	Septin-2	−1.414	0.0109
	Q5BJZ3	*Nnt*	Nicotinamide nucleotide transhydrogenase OS	−1.422	0.013
	P34058	*Hsp90ab1*	Heat shock protein HSP 90-beta	−1.254	0.0137
	A0A140TAI3	*Hnrnpc*	Heterogeneous nuclear ribonucleoprotein C, isoform CRA_b	−1.504	0.0144
	Q5RKI0	*Wdr1*	WD repeat-containing protein 1	−1.349	0.0146
	Q5BJU0	*Rras2*	RAS-related 2	−1.229	0.0147
	F1MAA7	*Lamc1*	Laminin subunit gamma 1	−1.413	0.0152
	P19511	*Atp5pb*	ATP synthase F(0) complex subunit B1, mitochondrial	−1.308	0.0152
	Q6P502	*Cct3*	T-complex protein 1 subunit gamma	−1.634	0.0152
	P10860	*Glud1*	Glutamate dehydrogenase 1, mitochondrial	−1.256	0.0162
	A0A0G2JSS9	*Atl3*	Atlastin-3	−1.198	0.0196
HEART	P57093	*Phyh*	Phytanoyl-CoA dioxygenase, peroxisomal	1.152	0.026
	A0A0G2K5P8	*Glrx3*	Glutaredoxin-3	−1.106	0.0263
	Q5PQN9	*Mrpl38*	39S ribosomal protein L38, mitochondrial	−1.256	0.0281

FC, fold change. *t*-test or Wilcoxon test of pairwise comparisons was performed depending on each protein’s distribution. * *p* < 0.02 was considered statistically significant.

## Data Availability

Data are available via ProteomeXchange (http://proteomecentral.proteomexchange.org/cgi/GetDataset, accessed on 10 January 2022) with identifier PXD018885.

## Supplementary Materials

The following are available online at https://www.mdpi.com/article/10.3390/nu14051047/s1: Figure S1. Thirty-six Wistar rats were divided into six groups of six animals each (three males and three females). Group 1: standard chow diet (SCD), Group 2: high-fat diet (HFD), Group 3: high-fat diet + red-fleshed apple (HFD + R), Group 4: high-fat diet + white-fleshed apple (HFD + W), Group 5: high-fat diet + *Aronia* (anthocyanin-rich extract; HFD + A); and Group 6: high-fat diet + atorvastatin (HFD + Atorv); Figure S2. Venn diagram showing the significant protein changes after HFD + W versus HFD in the aorta and B) heart tissue. Proteins coloured in green indicate decreased expression, and proteins coloured in red indicate increased expression compared to expression in the HFD group; Figure S3. Summary of the main findings in the aorta and heart proteome after supplementation with red-fleshed apple, white-fleshed apple, or anthocyanin-rich extract diets; Table S1. Phenolic composition of the main phenolics (µg phenolic/day/rat) in the white-fleshed apple snack, red-fleshed apple snack, and *Aronia* extract infusion; Table S2. Nutrient composition of each diet used in the study; Table S3. Biochemical parameters of rat plasma from de different groups studied; Table S4. Animal performance (mean ± standard deviation) according to diet treatment (*n* = 3 females + *n* = 3 males; global *n* = 6); Table S5. Aorta proteome; Table S6. Heart proteome; Table S7. Proteome changes on aorta and heart tissue of high-fat diet versus standard chow died; Table S8. Proteome changes on aorta or heart tissue of high-fat diet + atorvastatin versus high-fat diet.

## Appendix A. Material and Methods

### Appendix A.1. Proteomic Analysis

#### Appendix A.1.1. Protein Extraction and Quantification

First, samples were frozen with liquid nitrogen. Second, the samples were mixed with 1 mL of a radio-immunoprecipitation buffer and homogenized completely with a BlueBender (Next Advance, Inc., New York, NY, USA) using frozen/drawn cycles. Third, the samples were agitated for 1 h at 4 °C and centrifuged (13,000× *g*). Fourth, after centrifugation, the samples were sonicated with a 30 s pulse at 50% amplitude. Fifth, the samples were then centrifuged at 13,000× *g* for 15 min at 4 °C and supernatants were collected for protein precipitation with the addition of 10% trichloroacetic acid (TCA)/acetone. Sixth, the protein pellets were re-suspended in 6 M urea/50 mM ammonium bicarbonate solution and quantified by Bradford’s method.

#### Appendix A.1.2. Offgel-Nano LC-(Orbitrap) MS/MS Analysis

Fraction 1 (F1) was mixed with F7, F2 was mixed with F8, and so on until all fractions were mixed accordingly. In total 6 fractions were obtained and separated onto a C-18 reversed-phase nano-column (75 μm I.D.; 15 cm length; 3 μm particle diameter, Nikkyo Technos Co. Ltd., Tokyo, Japan) on an EASY-II nanoLC from Thermo Fisher Scientific (Waltham, MA, USA).

### Appendix A.2. Statistical Analysis

#### Appendix A.2.1. Data Pre-Processing

Ratios have the disadvantage of showing a non-intuitive scale. Thus, those proteins with a two-fold increase in rat aorta or heart tissue samples will have an HFD/SCD ratio of 2, whereas those with a two-fold decrease will have an HFD/SCD ratio of 0.5. The use of a log2 scale has the advantage of producing a continuous spectrum of values and treating a two-fold change in a similar fashion. Hence, a two-fold increase in the patients will be given by log2(2) = +1, whereas a two-fold decrease will be given by log2(1/2) = −1.

#### Appendix A.2.2. Multivariate Statistical Analysis

##### Unsupervised Methods

Unsupervised methods were initially applied to identify trends, groupings, and outliers. These methods work on unlabeled data, that is, they do not incorporate information such as a sample class (Y: treatments/controls). PCA is a projection method that summarizes the multivariate data (X) in a small number of principal components (which are linear combinations of the original variables X) based on the largest variation in the dataset. In the PCA scores plot, each dot represents the complete proteomic profile of one sample.

On the other hand, HCA clusters observations based on the similarity of their proteomic profiles and results are usually visualized as dendrograms and heat maps. For a comparative analysis across different proteins, data were standardized as z-scores across samples for each protein before clustering so that the mean was 0 and the standard deviation was 1. The standardized matrix was used in unsupervised HCA for samples and proteins using Euclidean-based distances from which hierarchical clusters were generated using a Ward-linkage.

##### Supervised Methods

Supervised methods incorporate additional information about the samples into the models to identify variation in the data that is correlated with the phenotypic response variables. Similar to PCA, PLS is a projection method that captures in its components the maximum covariance between the data (X) and the variable of interest (Y: response/class/phenotype). It is a multivariate regression technique to predict the response variable (Y) from linear combinations of the original variables (components). To evaluate the performance of each model, the goodness of fit (R2X) and the predictive performance (Q2Y), which relate to the explained and predicted variance, respectively, were calculated. The R2X always increases with the number of components, from 0, indicating that no variation in the data is modeled, to 1, where all the variation is modeled. On the other hand, Q2Y varies from −∞, which means that your model is not all predictive or is overfitted, to 1, which reflects a perfect predictive precision. Unlike R2X, it increases with the number of components but at some point it falls, indicating the no more components should be added. The difference between Q2Y and R2X is a rough measure of overfitting.

The importance of each individual X-variable on the model was estimated the Variable Importance for the Projection (VIP), which is a weighted sum of squares (SS) of the PLS weights, wa, k, with the weights calculated from the amount of Y-variance of each PLS component.

OPLS is similar to PLS but incorporates an orthogonal signal correction filter to improve interpretation, although it has the same predictive performance as PLS. It works by decomposing the data (X) in the so-called predictive component, related to the response variable Y, and the orthogonal components, containing the non-related information to the response. In order to assess the significance of class discrimination, a permutation test was performed.

In order to avoid overfitting, model validity was established by permutation testing (1000 permutations). It consists of comparing the Q2Y obtained for the original dataset with the distribution of Q2Y values calculated when the original Y values are randomly assigned to the individuals. Then, the position of the Q2Y for the original model in the distribution of Q2Y values obtained from the permutations is used to calculate a *p*-value to estimate the significance of the OPLS model.

In both PLS and OPLS methods, the response variable can be continuous or categorical. In the latter case, the term discriminant analysis (DA) is used and the response variable refers to the class membership. In this case, the objective is to discriminate/classify two or more classes and investigate the causes for class separation (in our case proteins that are in higher/lower concentration in patients compared to controls).

A tool for visualization and interpretation of multivariate classification OPLS-DA models is the S-plot. It visualizes both the covariance (p[1]), also called model loadings, and correlation (p(corr)[1]):

The p[1] axis describes the magnitude of each variable in X, whereas the p(corr)[1] axis represents the reliability of each variable in X. Biomarkers should have high reliability. However, peaks with high reliability but low magnitude/intensity are close to the noise level and there is a high risk for spurious correlations. Therefore, ideal biomarkers have high magnitude and high reliability and can be easily identified by both extremes of the S-plot.

## References

[1] Roth G.A., Johnson C., Abajobir A., Abd-Allah F., Abera S.F., Abyu G., Ahmed M., Aksut B., Alam T., Alam K. (2017). Global, Regional, and National Burden of Cardiovascular Diseases for 10 Causes, 1990 to 2015. J. Am. Coll. Cardiol..

[2] Warburton D.E.R., Bredin S.S.D. (2017). Health benefits of physical activity: A systematic review of current systematic reviews. Curr. Opin. Cardiol..

[3] Warburton D.E.R., Bredin S.S.D. (2016). Reflections on Physical Activity and Health: What Should We Recommend?. Can. J. Cardiol..

[4] Doughty K.N., Del Pilar N.X., Audette A., Katz D.L. (2017). Lifestyle Medicine and the Management of Cardiovascular Disease. Curr. Cardiol. Rep..

[5] Medina-Remón A., Kirwan R., Lamuela-Raventós R.M., Estruch R. (2018). Dietary patterns and the risk of obesity, type 2 diabetes mellitus, cardiovascular diseases, asthma, and neurodegenerative diseases. Crit. Rev. Food Sci. Nutr..

[6] Wang N., Jiang S., Zhang Z., Fang H., Xu H., Wang Y., Chen X. (2018). Malus sieversii: The origin, flavonoid synthesis mechanism, and breeding of red-skinned and red-fleshed apples. Hortic. Res..

[7] Sandoval-Ramírez B.A., Catalán Ú., Calderón-Pérez L., Companys J., Pla-Pagà L., Ludwig I.A., Romero M.P., Solà R. (2020). The effects and associations of whole-apple intake on diverse cardiovascular risk factors. A narrative review. Crit. Rev. Food Sci. Nutr..

[8] Patocka J., Bhardwaj K., Klimova B., Nepovimova E., Wu Q., Landi M., Kuca K., Valis M., Wu W. (2020). Malus domestica: A review on nutritional features, chemical composition, traditional and medicinal value. Plants.

[9] Hyson D.A. (2011). A comprehensive review of apples and apple components and their relationship to human health. Adv. Nutr..

[10] Kalinowska M., Bielawska A., Lewandowska-Siwkiewicz H., Priebe W., Lewandowski W. (2014). Apples: Content of phenolic compounds vs. variety, part of apple and cultivation model, extraction of phenolic compounds, biological properties. Plant Physiol. Biochem..

[11] Stirpe M., Palermo V., Bianchi M.M., Silvestri R., Falcone C., Tenore G., Novellino E., Mazzoni C. (2017). Annurca apple (M. pumila Miller cv Annurca) extracts act against stress and ageing in S. cerevisiae yeast cells. BMC Complement. Altern. Med..

[12] Manzano M., Giron M.D., Vilchez J.D., Sevillano N., El-Azem N., Rueda R., Salto R., Lopez-Pedrosa J.M. (2016). Apple polyphenol extract improves insulin sensitivity in vitro and in vivo in animal models of insulin resistance. Nutr. Metab..

[13] Yanni A.E., Efthymiou V., Lelovas P., Agrogiannis G., Kostomitsopoulos N., Karathanos V.T. (2015). Effects of dietary Corinthian currants (Vitis vinifera L., var. Apyrena) on atherosclerosis and plasma phenolic compounds during prolonged hypercholesterolemia in New Zealand White rabbits. Food Funct..

[14] Espley R.V., Butts C.A., Laing W.A., Martell S., Smith H., McGhie T.K., Zhang J., Paturi G., Hedderley D., Bovy A. (2014). Dietary flavonoids from modified apple reduce inflammation markers and modulate gut microbiota in mice. J. Nutr..

[15] Bars-Cortina D., Macià A., Iglesias I., Romero M.P., Motilva M.J. (2017). Phytochemical Profiles of New Red-Fleshed Apple Varieties Compared with Traditional and New White-Fleshed Varieties. J. Agric. Food Chem..

[16] Barnett M.P.G., Young W., Armstrong K., Brewster D., Cooney J.M., Ellett S., Espley R.V., Laing W., Maclean P., McGhie T. (2021). A polyphenol enriched variety of apple alters circulating immune cell gene expression and faecal microbiota composition in healthy adults: A randomized controlled trial. Nutrients.

[17] Zhao Y., Qu H., Wang Y., Xiao W., Zhang Y., Shi D. (2020). Small rodent models of atherosclerosis. Biomed. Pharmacother..

[18] Reagan-Shaw S., Nihal M., Ahmad N. (2008). Dose translation from animal to human studies revisited. FASEB J..

[19] Kilkenny C., Parsons N., Kadyszewski E., Festing M.F.W., Cuthill I.C., Fry D., Hutton J., Altman D.G. (2009). Survey of the Quality of Experimental Design, Statistical Analysis and Reporting of Research Using Animals. PLoS ONE.

[20] Kilkenny C., Browne W.J., Cuthill I.C., Emerson M., Altman D.G. (2012). Improving Bioscience Research Reporting: The ARRIVE Guidelines for Reporting Animal Research. Vet. Clin. Pathol..

[21] Aebersold R., Burlingame A.L., Bradshaw R.A. (2013). Western blots versus selected reaction monitoring assays: Time to turn the tables?. Mol. Cell. Proteom..

[22] (2013). Method of the year 2012. Nat. Methods.

[23] Perez-Riverol Y., Csordas A., Bai J., Bernal-Llinares M., Hewapathirana S., Kundu D.J., Inuganti A., Griss J., Mayer G., Eisenacher M. (2019). The PRIDE database and related tools and resources in 2019: Improving support for quantification data. Nucleic Acids Res..

[24] Blum A., Shamburek R. (2009). The pleiotropic effects of statins on endothelial function, vascular inflammation, immunomodulation and thrombogenesis. Atherosclerosis.

[25] Chu P.H., Chen J. (2011). The novel roles of four and a half LIM proteins 1 and 2 in the cardiovascular system. Chang Gung Med. J..

[26] Yuste S., Ludwig I.A., Romero M.P., Piñol-Felis C., Catalán Ú., Pedret A., Valls R.M., Fernández-Castillejo S., Motilva M.J., Macià A. (2021). Metabolic Fate and Cardiometabolic Effects of Phenolic Compounds from Red-Fleshed Apple in Hypercholesterolemic Rats: A Comparative Study with Common White-Fleshed Apple. The AppleCOR Study. Mol. Nutr. Food Res..

[27] Liu X., Xu X., Shang R., Chen Y. (2018). Asymmetric dimethylarginine (ADMA) as an important risk factor for the increased cardiovascular diseases and heart failure in chronic kidney disease. Nitric Oxide Biol. Chem..

[28] Sozański T., Kucharska A.Z., Wiśniewski J., Fleszar M.G., Rapak A., Gomułkiewicz A., Dzięgiel P., Magdalan J., Nowak B., Szumny D. (2019). The iridoid loganic acid and anthocyanins from the cornelian cherry (Cornus mas L.) fruit increase the plasma L-arginine/ADMA ratio and decrease levels of ADMA in rabbits fed a high-cholesterol diet. Phytomedicine.

[29] Weiss N., Zhang Y.Y., Heydrick S., Bierl C., Loscalzo J. (2001). Overexpression of cellular glutathione peroxidase rescues homocyst(e)ine-induced endothelial dysfunction. Proc. Natl. Acad. Sci. USA.

[30] Blankenberg S., Rupprecht H.J., Bickel C., Torzewski M., Hafner G., Tiret L., Smieja M., Cambien F., Meyer J., Lackner K.J. (2003). Glutathione Peroxidase 1 Activity and Cardiovascular Events in Patients with Coronary Artery Disease. N. Engl. J. Med..

[31] Haidar B., Kiss R.S., Sarov-Blat L., Brunet R., Harder C., McPherson R., Marcel Y.L. (2006). Cathepsin D, a lysosomal protease, regulates ABCA1-mediated lipid efflux. J. Biol. Chem..

[32] Wu Y., Potempa L.A., El Kebir D., Filep J.G. (2015). C-reactive protein and inflammation: Conformational changes affect function. Biol. Chem..

[33] Hage F.G. (2014). C-reactive protein and Hypertension. J. Hum. Hypertens..

[34] McGrath F.D.G., Brouwer M.C., Arlaud G.J., Daha M.R., Hack C.E., Roos A. (2006). Evidence That Complement Protein C1q Interacts with C-Reactive Protein through Its Globular Head Region. J. Immunol..

[35] Hertle E., Stehouwer C.D.A., van Greevenbroek M.M.J. (2014). The complement system in human cardiometabolic disease. Mol. Immunol..

[36] Vlaicu R., Rus H.G., Niculescu F., Cristea A. (1985). Immunoglobulins and complement components in human aortic atherosclerotic intima. Atherosclerosis.

[37] Vlaicu S.I., Tatomir A., Rus V., Mekala A.P., Mircea P.A., Niculescu F., Rus H. (2016). The role of complement activation in atherogenesis: The first 40 years. Immunol. Res..

[38] Hazarika S., Angelo M., Li Y., Aldrich A.J., Odronic S.I., Yan Z., Stamler J.S., Annex B.H. (2008). Myocyte specific overexpression of myoglobin impairs angiogenesis after hind-limb ischemia. Arterioscler. Thromb. Vasc. Biol..

[39] Merx M.W., Flögel U., Stumpe T., Gödecke A., Decking U.K.M., Schrader J. (2001). Myoglobin facilitates oxygen diffusion. FASEB J..

[40] Hermann M., Flammer A., Lscher T.F. (2006). Nitric Oxide in Hypertension. J. Clin. Hypertens..

[41] Hellman N.E., Gitlin J.D. (2002). Ceruloplasmin metabolism and function. Annu. Rev. Nutr..

[42] Grammer T.B., Kleber M.E., Silbernagel G., Pilz S., Scharnagl H., Lerchbaum E., Tomaschitz A., Koenig W., März W. (2014). Copper, ceruloplasmin, and long-term cardiovascular and total mortality (The Ludwigshafen Risk and Cardiovascular Health Study). Free Radic. Res..

[43] Mänttäri M., Manninen V., Hurrunen J.K., Palosuo T., Ehnholm C., Heinonen O.P., Frick M.H. (1994). Serum ferritin and ceruloplasmin as coronary risk factors. Eur. Heart J..

[44] Wang W., Knovich M.A., Coffman L.G., Torti F.M., Torti S.V. (2010). Serum ferritin: Past, present and future. Biochim. Biophys. Acta Gen. Subj..

[45] Shipra, Gupta B.K., Solanki R., Punia H., Agarwal V., Kaur J., Shukla A. (2014). Relationship of lipid profile and serum Ferritin levels with acute myocardial infarction. J. Clin. Diagnostic Res..

[46] Pedret A., Catalán Ú., Fernández-Castillejo S., Farràs M., Valls R.M., Rubió L., Canela N., Aragonés G., Romeu M., Castañer O. (2015). Impact of virgin olive oil and phenol-enriched virgin olive oils on the HDL proteome in hypercholesterolemic subjects: A double blind, randomized, controlled, cross-over clinical trial (VOHF study). PLoS ONE.

[47] Yamada K., Aiba K., Kitaura Y., Kondo Y., Nomura N., Nakamura Y., Fukushi D., Murayama K., Shimomura Y., Pitt J. (2015). Clinical, biochemical and metabolic characterisation of a mild form of human short-chain enoyl-CoA hydratase deficiency: Significance of increased n-acetyl-s-(2-carboxypropyl)cysteine excretion. J. Med. Genet..

[48] Du J., Li Z., Li Q.Z., Guan T., Yang Q., Xu H., Pritchard K.A., Camara A.K.S., Shi Y. (2013). Enoyl coenzyme a hydratase domain-containing 2, a potential novel regulator of myocardial ischemia injury. J. Am. Heart Assoc..

[49] Hewawasam R.P., Liu D., Casarotto M.G., Board P.G., Dulhunty A.F. (2016). The GSTM2 C-Terminal domain depresses contractility and Ca2+ transients in neonatal rat ventricular cardiomyocytes. PLoS ONE.

[50] Seidah N.G., Poirier S., Denis M., Parker R., Miao B., Mapelli C., Prat A., Wassef H., Davignon J., Hajjar K.A. (2012). Annexin A2 is a natural extrahepatic inhibitor of the PCSK9-induced LDL receptor degradation. PLoS ONE.

[51] Poirier S., Mayer G., Benjannet S., Bergeron E., Marcinkiewicz J., Nassoury N., Mayer H., Nimpf J., Prat A., Seidah N.G. (2008). The proprotein convertase PCSK9 induces the degradation of low density lipoprotein receptor (LDLR) and its closest family members VLDLR and ApoER2. J. Biol. Chem..

[52] Jitrapakdee S., Wallace J.C. (1999). Structure, function and regulation of pyruvate carboxylase. Biochem. J..

[53] Gambaryan S., Kobsar A., Rukoyatkina N., Herterich S., Geiger J., Smolenski A., Lohmann S.M., Walter U. (2010). Thrombin and collagen induce a feedback inhibitory signaling pathway in platelets involving dissociation of the catalytic subunit of protein kinase a from an NFκB-IκB complex. J. Biol. Chem..

[54] Huang X., Jin Y., Zhou D., Xu G., Huang J., Shen L. (2015). IQGAP1 modulates the proliferation and migration of vascular smooth muscle cells in response to estrogen. Int. J. Mol. Med..

[55] Haase M., Fitze G. (2016). HSP90AB1: Helping the good and the bad. Gene.

[56] Chadli A., Graham J.D., Abel M.G., Jackson T.A., Gordon D.F., Wood W.M., Felts S.J., Horwitz K.B., Toft D. (2006). GCUNC-45 Is a Novel Regulator for the Progesterone Receptor/hsp90 Chaperoning Pathway. Mol. Cell. Biol..

[57] Zhu J., Cole F., Woo-Rasberry V., Fang X.R., Chiang T.M. (2007). Type I and type III collagen-platelet interaction: Inhibition by type specific receptor peptides. Thromb. Res..

[58] Huang L., Li L., Yang T., Li W., Song L., Meng X., Gu Q., Xiong C., He J. (2018). Transgelin as a potential target in the reversibility of pulmonary arterial hypertension secondary to congenital heart disease. J. Cell. Mol. Med..

[59] Hailstones D.L., Gunning P.W. (1990). Characterization of human myosin light chains 1sa and 3nm: Implications for isoform evolution and function. Mol. Cell. Biol..

[60] Munjas J., Sopić M., Spasojević-Kalimanovska V., Kalimanovska-Oštrić D., Anđelković K., Jelić-Ivanović Z. (2017). Association of adenylate cyclase-associated protein 1 with coronary artery disease. Eur. J. Clin. Investig..

[61] Jang H.D., Lee S.E., Yang J., Lee H.C., Shin D., Lee H., Lee J., Jin S., Kim S., Lee S.J. (2020). Cyclase-associated protein 1 is a binding partner of proprotein convertase subtilisin/kexin type-9 and is required for the degradation of low-density lipoprotein receptors by proprotein convertase subtilisin/kexin type-9. Eur. Heart J..

[62] Son M., Diamond B., Santiago-Schwarz F. (2015). Fundamental role of C1q in autoimmunity and inflammation. Immunol. Res..

[63] Harboe M., Johnson C., Nymo S., Ekholt K., Schjalm C., Lindstad J.K., Pharo A., Hellerud B.C., Ekdahl K.N., Mollnes T.E. (2017). Properdin binding to complement activating surfaces depends on initial C3b deposition. Proc. Natl. Acad. Sci. USA.

[64] Aguilera J.M. (2019). The food matrix: Implications in processing, nutrition and health. Crit. Rev. Food Sci. Nutr..

[65] Monfoulet L.E., Buffière C., Istas G., Dufour C., Le Bourvellec C., Mercier S., Bayle D., Boby C., Remond D., Borel P. (2020). Effects of the apple matrix on the postprandial bioavailability of flavan-3-ols and nutrigenomic response of apple polyphenols in minipigs challenged with a high fat meal. Food Funct..

