# Supplementary material for: Red-Fleshed Apples Rich in Anthocyanins and White-Fleshed Apples Modulate the Aorta and Heart Proteome in Hypercholesterolaemic Rats: The AppleCOR Study
Source: Nutrients. 2022 Feb 28;14(5):1047. doi: 10.3390/nu14051047 (PMC8912372; doi:10.3390/nu14051047)
Supplement: Supplementary file 1 [file nutrients-14-01047-s001.zip › Supplementary Figure S1. Study design_rats_AppleCor.pdf]

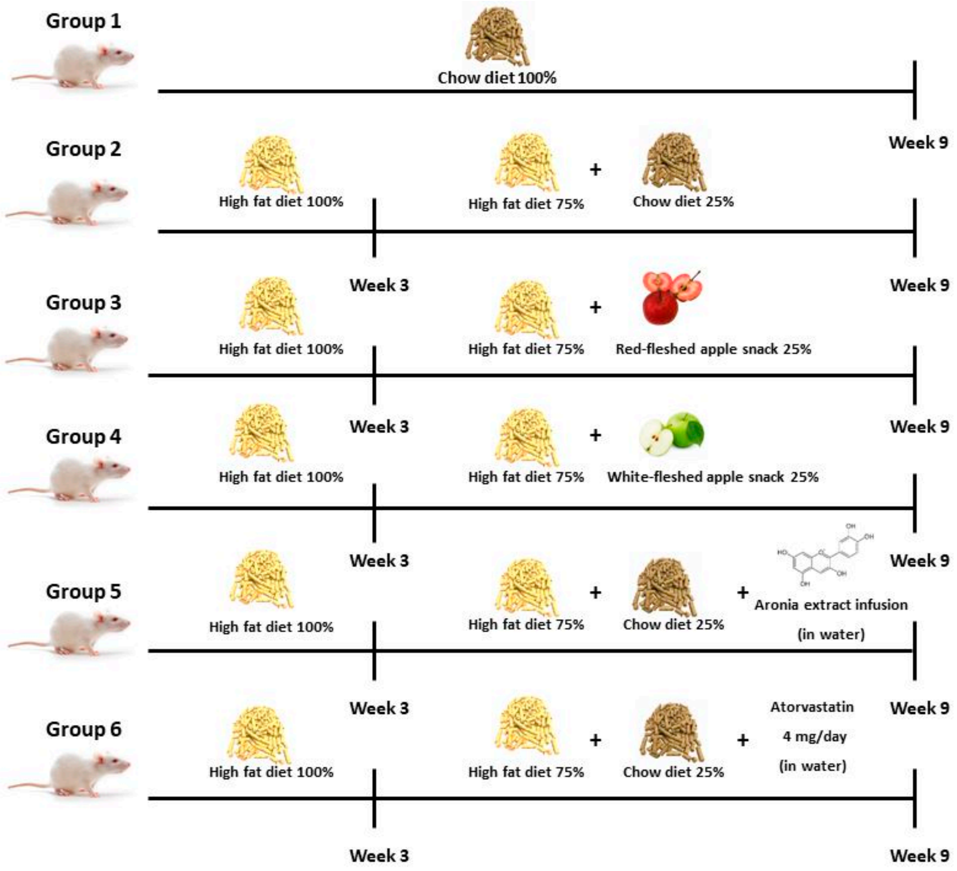

Figure S1. Thirty-six Wistar rats were divided into 6 groups of 6 animals each (3 males and 3 females). Group 1: standard chow diet (SCD), Group 2: high-fat diet (HFD), Group 3: high-fat diet + red-fleshed apple (HFD + R), Group 4: high-fat diet + white-fleshed apple (HFD + W), Group 5: high-fat diet + Aronia (anthocyanin-rich extract; HFD + A); and Group 6: high-fat diet + Atorvastatin (HFD + Atorv).
