# Supplementary material for: Red-Fleshed Apples Rich in Anthocyanins and White-Fleshed Apples Modulate the Aorta and Heart Proteome in Hypercholesterolaemic Rats: The AppleCOR Study
Source: Nutrients. 2022 Feb 28;14(5):1047. doi: 10.3390/nu14051047 (PMC8912372; doi:10.3390/nu14051047)
Supplement: Supplementary file 1 [file nutrients-14-01047-s001.zip › Supplementary Figure S2.pdf]

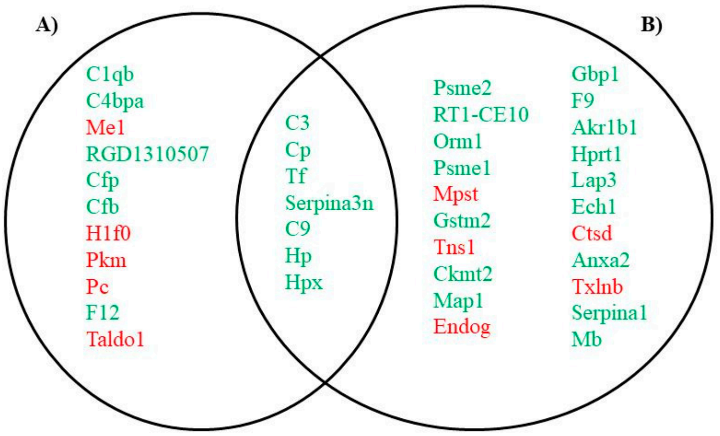

Figure S2. Venn diagram showing the significant protein changes after HFD + W versus HFD in the aorta and B) heart tissue. Protein coloured green indicate decreased expression, and protein coloured red indicate increased expression compared to expression in the HFD group.
