# Supplementary material for: Red-Fleshed Apples Rich in Anthocyanins and White-Fleshed Apples Modulate the Aorta and Heart Proteome in Hypercholesterolaemic Rats: The AppleCOR Study
Source: Nutrients. 2022 Feb 28;14(5):1047. doi: 10.3390/nu14051047 (PMC8912372; doi:10.3390/nu14051047)
Supplement: Supplementary file 1 [file nutrients-14-01047-s001.zip › Supplementary Table S1_Phenolic composition.pdf]

Supplementary Table S1. Phenolic composition of the main phenolics (µg phenolic/day/ rat) in the white-fleshed apple snack, red-fleshed apple snack, and Aronia extract infusion.

**Phenolic compounds**	**White-fleshed apple snack** (mean ± SD)	**Red-fleshed apple snack** (mean ± SD)	**Aronia extract infusion** (mean ± SD)
Cyanidin arabinoside	6.88 ± 6.12	167 ± 6.00	480 ± 18.0
Cyanidin 3-O-galactoside	21.8 ± 18.1	1690 ± 36.0	1426 ± 48.0
Total Anthocyanins	***28.6 ± 24.2***	***1857 ± 42.0***	***1906 ± 67.0***
Protocatechuic acid	n.d.	108 ± 67.3	462 ± 65.0
Coumaric acid hexoside	48.7 ± 7.03	48.7 ± 7.03	2.00 ± 0.00
Ferulic acid hexoside	67.3 ± 7.44	134 ± 16.4	5.00 ± 0.00
Vanillic acid	n.d.	n.d.	14.0 ± 1.00
Vanillic acid hexoside	58.2 ± 4.05	271 ± 7.24	138 ± 22.0
5-*O*-caffeoylquinic acid	1386 ± 28.08	5004 ± 174	814 ± 434
3-*O*-caffeoylquinic acid	n.d.	n.d.	362 ± 97.0
Gallic acid	n.d.	n.d.	36.0 ± 5.00
Gallic acid hexoside	n.d.	n.d.	25.0 ± 6.00
Caffeic acid	n.d.	n.d.	32.0 ± 1.00
Homogentisic acid	n.d.	n.d.	21.0 ± 7.00
Total Phenolic acids	***1837 ± 56.2***	***5566 ± 211***	***1911 ± 638***
Catechin	377 ± 25.3	n.d.	n.d.
Epicatechin	1867 ± 27.1	353 ± 49.3	12.0 ± 1.00
Dimer	3663 ± 121	438± 18.1	32.0 ± 7.00
Trimer	350 ± 51.6	82.0± 7.34	8.00 ± 1.00
Total Flavan-3-ols	***6259 ± 226***	***875 ± 74.9***	***52.0 ± 9.00***
Quercetin arabinoside	166 ± 24.3	232 ± 28.7	5.00 ± 1.00
Quercetin rhamnoside	230 ± 26.6	587 ± 59.7	n.d.
Quercetin glucoside	541 ± 81.0	279± 35.9	133 ± 11.0
Quercetin rutinoside	n.d.	n.d	87.0 ± 12.0
Total Flavonols	***938 ± 132***	***1098 ± 124.5***	***225 ± 24.0***
Eriodictyol hexoside	n.d.	26.4 ± 1.42	7.00 ± 2.00
Naringenin	n.d.	n.d.	n.d.
Total Flavanones	***n.d.***	***26.4 ± 1.42***	***7.00 ± 2.00***
Phloretin glucoside	247 ± 34.5	1371 ± 160	n.d.
Phloretin xylosyl glucoside	276 ± 17.6	739 ± 32.7	n.d.
Hydroxyphloretin xylosyl glucoside	14.2 ± 1.01	20.5 ± 1.52	n.d.
Total Dihydrochalcones	***536 ± 53.1***	***2130 ± 195***	***n.d.***
TOTAL PHENOLICS	***9598 ± 487***	***11552 ± 1420***	***4101 ± 740***

n.d., non-detected; SD, standard deviation.
