# Supplementary material for: Red-Fleshed Apples Rich in Anthocyanins and White-Fleshed Apples Modulate the Aorta and Heart Proteome in Hypercholesterolaemic Rats: The AppleCOR Study
Source: Nutrients. 2022 Feb 28;14(5):1047. doi: 10.3390/nu14051047 (PMC8912372; doi:10.3390/nu14051047)
Supplement: Supplementary file 1 [file nutrients-14-01047-s001.zip › Supplementary Table S2_Daily Dose Nutrients Rats Applecor.pdf]

Supplementary Table S2. Nutrient composition of each diet used in the study.

Nutrient information	Diet of different groups											
	**SCD**		**HFD**[b]		**HFD+R**[a]		**HFD+W**[a]		**HFD+A**[b, c]		**HFD+Atorv**[b, d]	
	Per 100 g	Per 20 g daily dose	Per 100 g	Per 20 g daily dose	Per 100 g	Per 20 g daily dose	Per 100 g	Per 20 g daily dose	Per 100 g	Per 20 g daily dose	Per 100 g	Per 20 g daily dose
**Protein**	14.3	2.9	16.6	3.3	13.7	2.7	13.6	2.7	16.6	3.3	16.6	3.3
**Carbohydrate**	48.0	9.6	47.2	9.4	58.6	11.7	58.3	11.7	51.7	10.3	47.2	9.4
**Fat**	4.0	0.8	16.9	3.4	16.1	3.2	16.1	3.2	16.9	3.4	16.9	3.4
**Kcal**	290.0	58.0	410.0	82.0	396.3	79.3	406.6	81.3	428.0	85.6	410.0	82.0
**Fiber**	4.12	0.82	1.03	0.21	4.13	0.83	3.47	0.69	1.03	0.21	1.03	0.21

SCD, standard chow diet; HFD, high-fat diet; HFD+R, HFD + red-fleshed apple; HFD+W, HFD + white-fleshed apple; HFD+A, HFD + Aronia extract infusion; HFD+Atorv, HFD + Atorvastatin

[a] Composed by 75% HFD + 25% apple snack (white or red-fleshed)

[b] Composed by 75% HFD + 25% SCD

[c] 100 g feed + 1 L of aronia extract infusion (daily dose: 20 g feed + 20 mL of aronia extract infusion)

[d] 4 mg Atorvastatin/day in the water
