# Supplementary material for: Red-Fleshed Apples Rich in Anthocyanins and White-Fleshed Apples Modulate the Aorta and Heart Proteome in Hypercholesterolaemic Rats: The AppleCOR Study
Source: Nutrients. 2022 Feb 28;14(5):1047. doi: 10.3390/nu14051047 (PMC8912372; doi:10.3390/nu14051047)
Supplement: Supplementary file 1 [file nutrients-14-01047-s001.zip › Supplementary Table S3.pdf]

Supplementary Table S3. Biochemical parameters of rat plasma from de different groups studied.

		TC (mg/dL)				TG (mg/dL)				HDLc (mg/dL)				non-HDLc(mg/dL)			
		mean	±	SD	*p*	mean	±	SD	*p*	mean	±	SD	*p*	mean	±	SD	*p*
SCD	Females (n=3)	69,22	±	7,72		56,39	±	29,30		52,46	±	2,83		16,76	±	6,14	
	Males (n=3)	73,47	±	13,30		140,53	±	90,85		54,59	±	7,27		18,88	±	7,09	
	Total (N=6)	71,35	±	10,00		98,46	±	75,95		53,53	±	5,07		17,82	±	6,05	
HFD	Females (n=3)	425,89	±	146,85	0,052	129,90	±	51,41	0,098	47,36	±	12,40	0,525	378,53	±	147,16	0.013[a]
	Males (n=3)	224,03	±	61,76	0.046[a]	151,45	±	42,95	0,860	36,30	±	11,92	0,086	187,73	±	52,82	0.029[a]
	Total (N=6)	324,96	±	149,58	0.009[a]	140,68	±	43,98	0,266	41,83	±	12,45	0,059	283,13	±	143,87	0.006[a]
HFD + red-fleshed apple	Females (n=3)	547,57	±	306,95	ns	215,52	±	143,73	ns	44,81	±	23,46	ns	502,76	±	288,71	ns
	Males (n=3)	232,54	±	33,63	ns	88,27	±	27,33	ns	40,55	±	9,37	ns	191,98	±	24,37	ns
	Total (N=6)	390,05	±	260,60	ns	151,90	±	115,84	ns	42,68	±	16,15	ns	347,37	±	250,11	ns
HFD + white-fleshed apple	Females (n=3)	476,80	±	189,68	ns	115,14	±	7,97	ns	33,46	±	15,60	ns	443,34	±	174,08	ns
	Males (n=3)	226,86	±	41,00	ns	91,52	±	29,80	ns	55,30	±	27,85	ns	171,57	±	39,64	ns
	Total (N=6)	351,83	±	183,86	ns	103,33	±	23,41	ns	44,38	±	23,47	ns	307,45	±	186,84	ns
HFD + Aronia	Females (n=3)	535,58	±	156,60	ns	211,68	±	68,65	ns	40,27	±	2,60	ns	495,31	±	154,01	ns
	Males (n=3)	236,02	±	66,82	ns	137,28	±	84,12	ns	43,39	±	13,37	ns	192,63	±	72,66	ns
	Total (N=6)	385,80	±	196,26	ns	174,48	±	79,85	ns	41,83	±	8,78	ns	343,97	±	197,70	ns
HFD + Atorvastatina	Females (n=3)	644,50	±	199,53	ns	184,82	±	82,79	ns	40,84	±	24,26	ns	603,66	±	179,80	ns
	Males (n=3)	220,03	±	50,16	ns	100,08	±	40,67	ns	33,75	±	15,69	ns	186,29	±	38,13	ns
	Total (N=6)	432,27	±	266,43	ns	142,45	±	74,55	ns	37,29	±	18,68	ns	394,98	±	256,46	ns

		ALT (U/L)				AST (U/L)				Insulin (ug/L)				Glucose (mg/dL)			
		mean	±	SD	*p*	mean	±	SD	*p*	mean	±	SD	*p*	mean	±	SD	*p*
SCD	Females (n=3)	43,33	±	16,17		170,00	±	88,61		0,46	±	0,10		221,67	±	18,56	
	Males (n=3)	44,33	±	10,07		179,00	±	1,73		0,84	±	0,13		264,00	±	39,85	
	Total (N=6)	43,83	±	12,06		174,50	±	56,27		0,61	±	0,23		242,83	±	36,20	
HFD	Females (n=3)	109,67	±	50,82	0,097	447,33	±	145,96	0.048[a]	1,24	±	0,66	0,113	225,33	±	22,94	0,840
	Males (n=3)	188,67	±	70,01	0.024[a]	313,67	±	76,83	0,093	5,27	±	0,34	<0.001[a]	210,67	±	11,24	0,090
	Total (N=6)	149,17	±	69,76	0.013[a]	380,50	±	127,45	0.005[a]	3,26	±	2,25	0.035[a]	218,00	±	18,04	0,164
HFD + red-fleshed apple	Females (n=3)	101,33	±	48,42	ns	380,00	±	93,74	ns	1,33	±	0,94	ns	278,33	±	62,32	ns
	Males (n=3)	209,67	±	40,38	ns	317,67	±	61,78	ns	3,22	±	0,34	ns	236,00	±	14,53	ns
	Total (N=6)	155,50	±	71,49	ns	348,83	±	78,79	ns	2,28	±	1,21	ns	257,17	±	46,65	ns
HFD + white-fleshed apple	Females (n=3)	107,33	±	61,44	ns	410,00	±	280,38	ns	1,07	±	0,74	ns	228,00	±	13,23	ns
	Males (n=3)	241,33	±	101,69	ns	493,67	±	218,53	ns	3,49	±	0,63	ns	227,67	±	13,65	ns
	Total (N=6)	174,33	±	105,04	ns	451,83	±	229,45	ns	2,52	±	1,44	ns	227,83	±	12,02	ns
HFD + Aronia	Females (n=3)	88,67	±	61,65	ns	358,33	±	197,45	ns	1,39	±	1,03	ns	260,33	±	44,29	ns
	Males (n=3)	246,00	±	23,52	ns	449,67	±	83,34	ns	2,08	±	2,24	0.029*	209,33	±	42,34	ns
	Total (N=6)	167,33	±	95,75	ns	404,00	±	144,48	ns	1,73	±	1,60	ns	234,83	±	47,77	ns
HFD + Atorvastatina	Females (n=3)	145,00	±	87,48	ns	512,33	±	130,22	ns	0,38	±	0,08	ns	238,33	±	32,13	ns
	Males (n=3)	262,33	±	118,56	ns	476,33	±	163,69	ns	3,31	±	0,30	ns	251,67	±	14,57	ns
	Total (N=6)	203,67	±	113,20	ns	494,33	±	133,75	ns	1,85	±	1,62	ns	245,00	±	23,48	ns

SCD, standard chow died; HFD, high fat diet; TC, total cholesterol; TG, triglycerides; HDLc, high-density lipoprotein cholesterol; nonHDLc, non-high-density lipoprotein cholesterol; ALT, alanine aminotransferase; AST, aspartate aminotransferase; ns, non significant

[a] HFD versus SCD student t-test
