# Supplementary material for: Red-Fleshed Apples Rich in Anthocyanins and White-Fleshed Apples Modulate the Aorta and Heart Proteome in Hypercholesterolaemic Rats: The AppleCOR Study
Source: Nutrients. 2022 Feb 28;14(5):1047. doi: 10.3390/nu14051047 (PMC8912372; doi:10.3390/nu14051047)
Supplement: Supplementary file 1 [file nutrients-14-01047-s001.zip › Supplementary Table S4_ Animal performance t-test.pdf]

Supplementary Table S4. Animal performance (mean ± standard deviation) according to diet treatment (n=3 females + n=3 males; global n=6)

	Increased weight (g)	*p*-value	Liquid intake (ml/day)	*p*-value	Food intake (g/day)	*p*-value
HFD	209 ± 87		36 ± 7.8		20 ± 2.7	
SCD	187 ± 95	0.680	31 ± 4.3	0.208	22 ± 3.8	0.412
HFD + R	196 ± 95	0.801	33 ± 11	0.553	19 ± 3.3	0.551
HFD + W	213 ± 104	0.948	38 ± 5.1	0.674	21 ± 2.9	0.642
HFD + A	227 ± 103	0.752	42 ± 3.8	0.521	18 ± 4.1	0.446
HFD + Atorv	229 ± 105	0.728	37 ± 2.8	0.805	19 ± 3.8	0.623

SCD, standard chow diet; HFD, high-fat diet; HFD+R, HFD + red-fleshed apple; HFD+W, HFD + white-fleshed apple; HFD+A, HFD + Aronia extract infusion; HFD+Atorv, HFD + Atorvastatin.

Data were analyzed using Student t-test comparing each treatment versus HFD.

* $p < 0.05$ was considered statistically significant.
