# Supplementary material for: Red-Fleshed Apples Rich in Anthocyanins and White-Fleshed Apples Modulate the Aorta and Heart Proteome in Hypercholesterolaemic Rats: The AppleCOR Study
Source: Nutrients. 2022 Feb 28;14(5):1047. doi: 10.3390/nu14051047 (PMC8912372; doi:10.3390/nu14051047)
Supplement: Supplementary file 1 [file nutrients-14-01047-s001.zip › Supplementary Table S7_HFD vs SCD.pdf]

Supplementary Table S7. Proteome changes on aorta and heart tissue of high-fat diet *versus* standard chow died.

Tissue	UniProt ID	Gene	Protein name	FC	*p*-value*
AORTA	P02680	*Fgg*	Fibrinogen gamma chain	2.228	< 0.001
	Q7TQ70	*Fga*	Ac1873	1.989	0.0010
	M0RBF1	*C3*	Complement C3	1.489	0.0019
	A0A0G2K162	*Epb41l2*	Erythrocyte membrane protein band 4.1-like 2	1.197	0.0022
	P49242	*Rps3a*	40S ribosomal protein S3a	1.302	0.0027
	Q9Z1Z9	*Pdlim7*	PDZ and LIM domain protein 7	1.239	0.0043
	A1L1M0	*Prkaca*	Protein kinase, cAMP-dependent, catalytic, alpha	1.240	0.0064
	D4A5E5	*Sgcb*	Sarcoglycan, beta	1.331	0.0085
	Q6P502	*Cct3*	T-complex protein 1 subunit gamma	1.622	0.0087
	G3V7N9	*C1qb*	Complement C1q subcomponent subunit B	2.001	0.0111
	P10860	*Glud1*	Glutamate dehydrogenase 1, mitochondrial	1.297	0.0118
	Q5RJR9	*Serpinh1*	Serine (Or cysteine) proteinase inhibitor, clade H, member 1, isoform CRA_b	1.093	0.0126
	D3ZFH5	*Itih2*	Inter-alpha-trypsin inhibitor heavy chain 2	1.698	0.0154
	P14046	*A1i3*	Alpha-1-inhibitor 3	1.494	0.0166
	B5DEZ8	*Plxdc2*	Plexin domain containing 2 (Predicted)	-1.122	0.0043
	Q5U2Q3	*N/A*	Ester hydrolase C11orf54 homolog	-1.426	0.0049
	M0RAM5	*Gpx1*	Glutathione peroxidase	-1.144	0.0071
	B5DEN5	*Eef1b2*	Eukaryotic translation elongation factor 1 beta 2	-1.260	0.0111
	D4A0T0	*Ndufb10*	NADH:ubiquinone oxidoreductase subunit B10	-1.397	0.0116
	P80254	*Ddt*	D-dopachrome decarboxylase	-1.466	0.0120
	P62775	*Mtpn*	Myotrophin	-1.416	0.0122
	Q6P686	*Ostf1*	Osteoclast-stimulating factor 1	-1.225	0.0152
	Q9WUC4	*Atox1*	Copper transport protein ATOX1	-1.819	0.0165
	A0A0G2K6J5	*Myl6*	Myosin light polypeptide 6	-1.631	0.0167
	O08557	*Ddah1*	N(G),N(G)-dimethylarginine dimethylaminohydrolase 1	-1.365	0.0190
HEART	P09605	*Ckmt2*	Creatine kinase S-type, mitochondrial	1.532	0.0015

P08010	*Gstm2*	Glutathione S-transferase Mu 2	1.386	0.0034
P16290	*Pgam2*	Phosphoglycerate mutase 2	1.260	0.0053
B4F789	*Apobec2*	Apolipoprotein B editing complex 2 (Predicted), isoform CRA_a	1.403	0.0055
Q62651	*Ech1*	Delta(3,5)-Delta(2,4)-dienoyl-CoA isomerase, mitochondrial	1.175	0.0083
B0BNE6	*Ndufs8*	NADH dehydrogenase (Ubiquinone) Fe-S protein 8 (Predicted), isoform CRA_a	1.427	0.0096
P00173	*Cyb5a*	Cytochrome b5	1.303	0.0100
Q9QZ76	*Mb*	Myoglobin	1.609	0.0100
D3ZPF0	*Fhl3*	Four and a half LIM domains 3	1.150	0.0139
E9PU42	*Vps26c*	Down syndrome critical region gene 3 (Predicted), isoform CRA_c	1.679	0.0152
A0A0G2JY31	*Serpina1*	Alpha-1-antiproteinase	1.234	0.0177
P35053	*Gpc1*	Glypican-1	1.291	0.0182
Q4V8F6	*Pcbp2*	Pcbp2 protein	-1.273	0.0022
P70623	*Fabp4*	Fatty acid-binding protein, adipocyte	-1.346	0.0157
F1LN42	*Tns1*	Tensin 1	-1.280	0.0176
P13086	*Suclg1*	Succinate--CoA ligase [ADP/GDP-forming] subunit alpha, mitochondrial	-1.288	0.0196
M0R7S5	*Plin4*	Perilipin 4	-1.497	0.0197

FC, fold change.
T-test or Wilcoxon test of pairwise comparisons was performed depending on each protein's distribution.
* $p<0.02$ was considered statistically significant
