# Supplementary material for: Red-Fleshed Apples Rich in Anthocyanins and White-Fleshed Apples Modulate the Aorta and Heart Proteome in Hypercholesterolaemic Rats: The AppleCOR Study
Source: Nutrients. 2022 Feb 28;14(5):1047. doi: 10.3390/nu14051047 (PMC8912372; doi:10.3390/nu14051047)
Supplement: Supplementary file 1 [file nutrients-14-01047-s001.zip › Supplementary Table S8_HFD+Atorv versus HFD.pdf]

Supplementary Table S8. Proteome changes on aorta or heart tissue of high-fat diet + Atorvastatina *versus* high-fat diet.

Tissue	UniProt ID	Gene	Protein name	FC	*p*-value*
AORTA	A0A0G2JUZ3	*Mypop*	Myb-related transcription factor, partner of profilin	1.331	0.0047
	Q9QZR6	*Sept9*	Septin-9	-1.238	0.0086
	A0A0G2JW88	*Map4*	Microtubule-associated protein	-1.582	0.0087
	Q9WUH4	*Fhl1*	Four and a half LIM domains protein 1	-1.465	0.0108
HEART	F1LN42	*Tns1*	Tensin 1	1.297	0.0008
	Q4V8F6	*Pcbp2*	Pcbp2 protein	1.245	0.0022
	P47942	*Dpysl2*	Dihydropyrimidinase-related protein 2	1.188	0.0022
	G3V8L3	*Lmna*	Lamin A, isoform CRA_b	1.267	0.0023
	M0RAM5	*Gpx1*	Glutathione peroxidase	1.177	0.0065
	P56571	*N/A*	ES1 protein homolog, mitochondrial	1.159	0.0148
	Q62651	*Ech1*	Delta(3,5)-Delta(2,4)-dienoyl-CoA isomerase, mitochondrial	-1.298	0.0012
	Q9QZ76	*Mb*	Myoglobin	-1.727	0.0027
	P08010	*Gstm2*	Glutathione S-transferase Mu 2	-1.354	0.0041
	P67779	*Phb*	Prohibitin	-1.142	0.0085
	P47727	*Cbr1*	Carbonyl reductase [NADPH] 1	-1.300	0.0130
	D3ZLT1	*Ndufb7*	NADH dehydrogenase (Ubiquinone) 1 beta subcomplex, 7 (Predicted)	-1.613	0.0131
	P25113	*Pgam1*	Phosphoglycerate mutase 1	-1.237	0.0148
	P19804	*Nme2*	Nucleoside diphosphate kinase B	-1.356	0.0152
	D3ZF77	*Akr1c15*	Aldo-keto reductase family 1 member C15	-1.235	0.0163
	P09605	*Ckmt2*	Creatine kinase S-type, mitochondrial	-1.499	0.0172

FC, fold change.
T-test or Wilcoxon test of pairwise comparisons was performed depending on each protein's distribution.
* $p<0.02$ was considered statistically significant
